# Early Sepsis Detection Using Heterogeneous Structured ICU Data with Explainable Deep Learning

**DOI:** 10.3390/s26123648

**Published:** 2026-06-08

**Authors:** Attaphongse Taparugssanagorn, Mariella Särestöniemi, Matti Hämäläinen, Jari Iinatti

**Affiliations:** 1 Faculty of Advanced Science and Technology, Asian Institute of Technology, Pathum Thani 12120, Thailand; attaphongset@ait.asia; 2 Health Science and Technology, Faculty of Medicine, University of Oulu, FI-90014 Oulu, Finland; 3 Centre for Wireless Communications, University of Oulu, FI-90014 Oulu, Finland; matti.hamalainen@oulu.fi (M.H.); jari.iinatti@oulu.fi (J.I.)

**Keywords:** explainable artificial intelligence (XAI), early sepsis prediction, ICU monitoring, PhysioNet Sepsis Challenge dataset, hybrid deep learning

## Abstract

Sepsis is life-threatening organ dysfunction caused by a dysregulated host response to infection, making early detection critical for improving outcomes in intensive care units (ICUs). This study presents a retrospective comparative evaluation of deep learning architectures for predicting sepsis up to 6 h before the PhysioNet/Computing in Cardiology 2019 Challenge onset label using hourly structured electronic health record (EHR) variables, including vital signs, laboratory measurements, and demographics. Evaluated architectures include Convolutional Neural Network (CNN), Long Short-Term Memory (LSTM), Gated Recurrent Unit (GRU), Bidirectional Long Short-Term Memory (Bi-LSTM), Temporal Convolutional Network (TCN), Transformer, and hybrid Convolutional Neural Network–Vision Transformer (CNN-ViT) models. Median imputation and class-weighted loss were applied to address missing values and severe class imbalance, while Shapley Additive Explanations (SHAP) and attention analyses were used as complementary interpretability approaches. Among the evaluated models, CNN-ViT achieved the strongest overall minority-class performance, with 88.25% accuracy, 0.7480 recall, a 0.454 F1-score, and a 0.48 area under the precision–recall curve (AUPRC), although the numerical gains over other advanced temporal and hybrid architectures were modest. Leave-one-unit-out evaluation further demonstrated relatively stable performance under internal distribution shifts. The results suggest that combining local feature extraction with temporal and attention-based modeling can improve early sepsis prediction from structured ICU data. However, the study represents a retrospective computational benchmark using a public dataset and does not constitute prospective clinical validation or real-world deployment assessment.

## 1. Introduction

Sepsis remains a leading cause of morbidity and mortality in intensive care units (ICUs) worldwide. Early detection is critical, as delayed recognition can result in rapid progression to septic shock and multi-organ failure. A systematic review by Islam et al. (2023) underscores the significance of machine learning (ML) and deep learning (DL) methods for sepsis detection and early prediction using electronic health record (EHR) data [1].

Conventional clinical detection relies on periodic monitoring and scoring systems, such as the Sequential Organ Failure Assessment (SOFA) score or the Systemic Inflammatory Response Syndrome (SIRS) criteria, which may fail to capture subtle temporal patterns in vital signs and laboratory values [2]. High-resolution time-series analyses of blood pressure (BP) and heart rate (HR) dynamics, for instance, have been shown to provide independent predictors of sepsis, even after adjusting for standard clinical measurements and patient demographics [3]. These findings suggest that continuous heterogeneous structured ICU monitoring, coupled with advanced time-series modeling, can enable the earlier and more accurate identification of patients at risk for sepsis.

Recent advances in ML, including Recurrent Neural Networks (RNNs), such as Long Short-Term Memory (LSTM) and Gated Recurrent Units (GRUs), as well as Transformer architectures, have enabled the extraction of complex temporal dependencies from continuous physiological data. By integrating heterogeneous physiological time-series measurements, including HR, BP, oxygen saturation (SpO_2_), respiratory rate (RR), temperature, and selected laboratory measurements, such as white blood cell counts and lactate levels, predictive models can achieve higher accuracy for early sepsis detection than approaches relying on isolated variable groups [4,5,6].

Furthermore, explainable artificial intelligence (XAI) methods, including Shapley Additive Explanations (SHAP) and attention analysis, help to identify influential variables and temporal regions in model predictions. These tools can support more transparent reviews of model behavior in high-risk ICU prediction tasks [4,6].

### 1.1. Related Existing Works and Research Gap

Over the past decade, numerous studies have investigated the application of ML and DL methods for early sepsis detection and mortality prediction in ICU settings. For example, ref. [5] developed a framework using structured EHR data, demonstrating that predictive models can detect sepsis onset earlier than conventional scoring systems, such as SOFA and SIRS. While promising, this approach relied solely on structured features and did not leverage unstructured clinical notes or high-resolution physiological signals. To address this, ref. [7] employed neural document embeddings to extract information from clinical notes, improving mortality prediction by capturing latent patterns absent from structured data. Later, ref. [6] combined structured and unstructured data, demonstrating that combining complementary clinical data sources can enhance the predictive accuracy, while emphasizing interpretability and applicability in resource-limited ICU environments. The present study focuses specifically on heterogeneous structured ICU EHR time-series data rather than fusion across distinct data types. Transformer-based hybrids and SHAP-based explanations are established methodological components; therefore, the research gap addressed here concerns their controlled comparison, combined feature-level and temporal interpretation, and robustness assessment in an ICU sepsis prediction benchmark, rather than architectural novelty alone.

Recent work has further emphasized the need to move beyond isolated model development toward clinically contextualized ICU prediction systems. The PhysioNet/Computing in Cardiology 2019 Challenge established a widely used benchmark for early sepsis prediction from routinely collected clinical time-series data [8]. Sepsis-specific deep learning studies include LiSep LSTM and approaches combining structured and unstructured clinical information [9,10]. The foundational and recent sequence modeling literature further motivates the comparison of recurrent, convolutional, Transformer, CNN–Transformer, and temporal attention architectures [11,12,13,14,15,16,17,18,19,20,21,22,23,24]. Broader advances in stereo segmentation, heterogeneous representation learning, biomedical segmentation, multimodal medicine, explainable healthcare AI, EHR foundation models, and efficient state-space sequence models provide additional methodological context for combining local feature extraction, global dependency modeling, heterogeneous structured data, and interpretability [25,26,27,28,29,30,31,32,33,34,35,36,37,38,39]. Class imbalance, evaluation metrics, F-measure interpretation, systematic sepsis outcome modeling, and feature-space analysis are also central to the evaluation strategy used in this study [40,41,42,43,44,45]. These studies motivate the present focus on structured ICU time-series benchmarking, local–global temporal modeling, and explicit interpretability analysis.

Despite these advances, key gaps remain. Most prior studies rely on retrospective datasets, limiting their generalizability and real-time applicability. Additionally, the integration of high-resolution physiological signals, such as ECG, blood pressure, and heart rate dynamics, has been limited. These signals can reveal early sepsis indicators that low-resolution measurements may miss [3,4]. Finally, while interpretability methods like SHAP and attention mechanisms have been applied, their clinical relevance is often evaluated qualitatively, leaving uncertainty about the clinically meaningful interpretation of model behavior and feature importance. These gaps motivate the need for a framework that systematically models heterogeneous structured ICU data with interpretable temporal learning for future AI-assisted clinical decision support research.

### 1.2. Our Contributions

Early sepsis prediction from ICU data remains challenging due to complex temporal dynamics, heterogeneous clinical variables, and severe class imbalance. Existing studies often focus on a single model family or lack systematic evaluation and interpretability. Accordingly, the contribution of this work is positioned as a systematic and transparent comparative investigation rather than as the proposal of an entirely new neural architecture. The study emphasizes reproducible evaluation, hybrid local–global temporal modeling, robustness under class imbalance and unit-level distribution shift, and explainability-oriented analysis for structured ICU time-series prediction. The individual model families considered here are established and well understood; the contribution of this study lies in a unified, controlled benchmark that compares these architectures under identical experimental conditions, integrates SHAP and attention-based interpretation, examines clinically meaningful feature importance patterns across competing models, and discusses dataset characteristics and deployment limitations for interpretable AI in critical care. This study makes the following contributions:

1. We investigate a hybrid deep learning framework that integrates Convolutional Neural Networks (CNNs), recurrent architectures (Long Short-Term Memory (LSTM), Gated Recurrent Unit (GRU), and Bidirectional Long Short-Term Memory (Bi-LSTM)), Temporal Convolutional Networks (TCNs), standard Transformer models, and a CNN–Vision Transformer (CNN-ViT) hybrid. Here, ViT refers to the Vision Transformer concept adapted to structured ICU time-series representations, where local CNN-derived feature patterns are combined with patch/embedding-based self-attention to model global dependencies across time and variables. The intended novelty lies not in introducing a completely new network family but in systematically evaluating how complementary local, sequential, and global temporal representations behave in multivariate ICU time-series data for predicting sepsis up to 6 h prior to onset.
2. We conduct the systematic benchmarking of multiple deep learning architectures under consistent preprocessing, training, and evaluation protocols using the PhysioNet/Computing in Cardiology 2019 Sepsis Challenge dataset and its annotation protocol. In contrast to prior studies that used varying experimental settings, this enables a controlled and fair comparison of temporal modeling approaches. Architectural settings, preprocessing assumptions, class imbalance handling, optimization choices, and validation procedures are specified to improve the experimental transparency and reproducibility. This design highlights the study’s main contribution as comprehensive benchmarking under a common experimental protocol, rather than architecture invention.
3. We develop an interpretable prediction framework that combines SHAP-based feature attribution with attention-based analysis. While many existing works treat deep learning models as black boxes, this approach provides both feature-level and temporal-level interpretability of model predictions. The combined explanation analysis is used to compare clinically meaningful feature importance patterns across model families, not to claim that SHAP or attention mechanisms are themselves new methods.
4. We evaluate model robustness under severe class imbalance and perform leave-one-unit-out cross-unit validation. Unlike the standard random splits used in prior work, this evaluation better reflects real-world generalization across different ICU units and patient populations. This robustness analysis is paired with an explicit discussion of dataset characteristics, limitations, and implications for the future deployment of interpretable AI systems in critical care.
5. We demonstrate that hybrid architectures, particularly CNN-ViT, a CNN-based local feature extractor combined with a ViT-inspired attention component for structured ICU time-series embeddings, achieve consistently strong predictive performance while maintaining interpretability and robustness across validation settings, highlighting their potential suitability for clinical decision support research following prospective validation.

### 1.3. Paper Organization

The remainder of this paper is organized as follows. Section 2 describes the dataset, including structured EHR features, physiological variables, laboratory measurements, demographic variables, data quality characteristics, and missingness patterns, along with the data preprocessing and feature extraction procedures. Section 3 details the proposed hybrid framework, describing the deep learning architectures employed, including 1D and 2D CNNs, LSTM, GRUs, standalone Transformer models, and hybrid architectures, such as CNN-LSTM and CNN-ViT. Here, CNN-ViT denotes a ViT-inspired patch/embedding-based attention variant adapted to multivariate ICU time–feature representations. This section also presents the heterogeneous structured ICU data modeling strategy and the XAI techniques used to facilitate the clinically meaningful interpretation of model behavior and feature importance. Section 4 outlines the experimental setup, including model training procedures, evaluation metrics, and validation protocols. Section 5 presents the results, including performance comparisons with baseline models and analyses of interpretability outcomes. Section 6 discusses the findings, emphasizing the clinical relevance, interpretability, and limitations of the proposed hybrid framework for the early detection of sepsis in the ICU. This section further discusses how heterogeneous structured ICU measurements and advanced deep learning architectures contribute to model robustness and interpretability, and how explainable AI techniques support the clinically meaningful interpretation of model predictions. Section 7 concludes the paper, summarizing key contributions and outlining potential directions for future work, including prospective validation, domain adaptation, model compression, potential relevance for real-time ICU monitoring scenarios, and extension to other critical conditions.

## 2. Materials and Methods

### 2.1. System Overview

**Figure 1:**
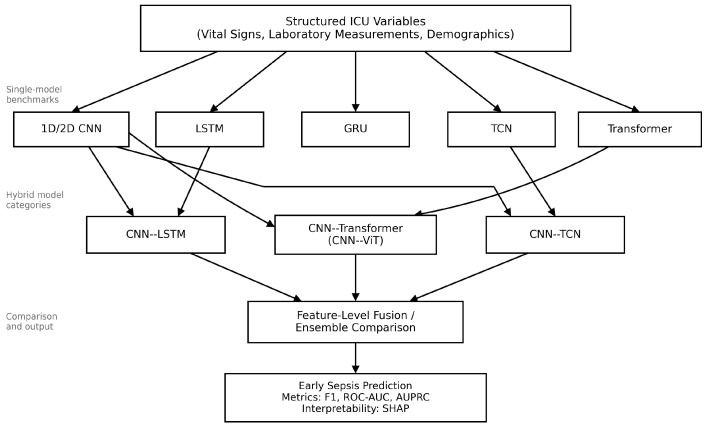
Overview of the proposed hybrid deep learning framework for early sepsis prediction using structured ICU variables. The enlarged schematic summarizes data preprocessing, model family benchmarking, hybrid architectures, ensemble comparison, explainability analysis, and evaluation outputs; connecting lines indicate workflow organization rather than architectural inheritance.

Figure 1 illustrates the proposed hybrid deep learning framework for early sepsis prediction in intensive care unit (ICU) patients. The system operates on structured electronic health record (EHR) variables available in the PhysioNet/Computing in Cardiology 2019 Sepsis dataset, including vital signs, laboratory measurements, and demographic information recorded as multivariate time-series data.

The visual organization of Figure 1 is intended to show the benchmarking workflow and model family categorization rather than architectural inheritance. The connecting lines indicate the direction of data processing and the grouping of related model classes within the evaluation pipeline. They should not be interpreted as one-to-one dependencies between adjacent models. In particular, the CNN-ViT branch denotes a hybrid local–global modeling category that combines convolutional feature extraction with Transformer-style attention, rather than a model derived from the GRU block shown in the same schematic tier.

These structured ICU variables serve as the primary input features for model training and prediction. Each patient trajectory is represented as a time–feature matrix and processed by multiple deep learning architectures, including 1D and 2D Convolutional Neural Networks (CNNs); recurrent models such as Long Short-Term Memory (LSTM), Gated Recurrent Units (GRUs), and Bidirectional Long Short-Term Memory (Bi-LSTM); Temporal Convolutional Networks (TCNs); and Transformer-based architectures. These models capture complementary temporal characteristics of the data, including short-term physiological fluctuations, sequential dependencies, and long-range temporal relationships.

Hybrid architectures are further constructed to leverage the strengths of different model families, including CNN-LSTM and CNN–Vision Transformer (CNN-ViT) models. In the CNN-ViT configuration, the multivariate time–feature matrix is interpreted as a two-dimensional representation and divided into patches to enable patch-based attention modeling. In this work, the ViT-inspired Transformer component is adapted for structured multivariate ICU temporal embeddings rather than image patches.

Predictions generated by individual and hybrid models are also combined using a weighted ensemble strategy to produce a comparative sepsis risk score, representing the probability of early sepsis onset within the predefined prediction horizon. The ensemble is used only as a benchmark for probability fusion and is not presented as a new model architecture.

Model performance is evaluated using accuracy, precision, recall, the F1-score, and the area under the precision–recall curve (AUPRC). To improve the transparency and interpretability, Shapley Additive Explanations (SHAP) are applied to analyze the contributions of individual clinical variables to model predictions.

To further assess model robustness and generalization, a leave-one-unit-out cross-unit validation strategy is employed using ICU unit identifiers within the dataset. This evaluation protocol simulates external validation by testing the model on data from a held-out ICU unit, enabling the analysis of performance under potential distribution shifts across clinical environments. Nevertheless, this analysis remains an internal retrospective robustness assessment based on a public benchmark dataset and should not be interpreted as prospective clinical validation or as evidence of deployment readiness in an independent hospital system.

### 2.2. Dataset Description

This study uses the PhysioNet/Computing in Cardiology 2019 Challenge sepsis dataset [8]. The dataset contains de-identified ICU patient records and is designed for the early prediction of sepsis onset. It includes longitudinal clinical time-series data from 40,336 critically ill patients and 40 structured predictor variables commonly monitored in critical care settings. These data comprise EHR variables such as vital signs (e.g., heart rate, blood pressure, respiratory rate, and oxygen saturation), laboratory measurements (e.g., white blood cell count and lactate levels), and patient demographic information (e.g., age and gender). Measurements are organized into hourly time steps, forming multivariate time-series suitable for machine learning analysis.

Sepsis labels follow the PhysioNet/Computing in Cardiology 2019 Sepsis Challenge annotation protocol, where an onset timestamp is provided to indicate when sepsis is considered to occur based on challenge-defined clinical criteria derived from electronic health record data. This protocol is based on a Sepsis-3–inspired definition, in which sepsis onset is identified retrospectively using indicators of suspected infection together with organ dysfunction signals approximating an increase in the Sequential Organ Failure Assessment (SOFA) score.

In this study, these annotations are used to formulate a prediction task for detecting sepsis up to 6 h prior to the annotated onset time. The onset timestamp serves as the reference anchor point for defining the prediction window and evaluating model performance.

Measurements reflect typical ICU monitoring practice, where vital signs are recorded frequently and laboratory tests are obtained periodically. This combination enables the dataset to capture both rapid physiological fluctuations and slower clinical trends that may precede sepsis onset.

In this work, the dataset is characterized as heterogeneous structured ICU EHR data, comprising vital signs, laboratory measurements, and demographic information. The dataset does not include additional data sources such as medical imaging, high-resolution waveform signals, or unstructured clinical notes; therefore, this terminology more precisely reflects the available structured clinical measurements while emphasizing their complementary physiological and laboratory information.

The dataset presents several challenges typical of real-world clinical data, including missing measurements, irregular sampling, and heterogeneity across patient populations and ICU units. These characteristics introduce variability and potential distribution shifts, making the dataset a realistic benchmark for evaluating machine learning models for early sepsis prediction.

Overall, the PhysioNet/Computing in Cardiology 2019 dataset provides a well-established benchmark for studying machine learning approaches to early sepsis detection and enables a systematic comparison across different model architectures and temporal modeling strategies.

The dataset is characterized explicitly as a multi-center ICU sensing and EHR benchmark. The full PhysioNet/Computing in Cardiology 2019 Challenge dataset contains longitudinal records from 40,336 critically ill patients collected from three independent hospital systems. Each patient record is represented as an hourly time series and contains 40 predictor variables together with the sepsis outcome label. The dataset therefore reflects real-world intensive care environments with heterogeneous patient populations, monitoring systems, laboratory workflows, documentation practices, and institutional care protocols.

The measurement modalities can be grouped into three clinically distinct categories. First, eight physiological monitoring variables are commonly acquired through bedside monitoring devices and physiological sensors: heart rate (HR), peripheral oxygen saturation (O_2_Sat), body temperature (Temp), systolic blood pressure (SBP), mean arterial pressure (MAP), diastolic blood pressure (DBP), respiratory rate (Resp), and end-tidal carbon dioxide (EtCO_2_). These variables represent continuous or near-continuous physiological observations generated by ICU monitoring systems, although the released challenge data are discretized into hourly records. Second, 26 laboratory variables are derived from clinical laboratory testing, including lactate, creatinine, bilirubin, glucose, white blood cell count, platelet count, blood urea nitrogen, bicarbonate, arterial oxygenation measurements, coagulation indicators, and other biochemical markers relevant to organ dysfunction and sepsis progression. Third, six demographic and administrative variables, namely age, gender, ICU unit identifiers, hospital admission time, and ICU length of stay, provide contextual patient-level information.

Unlike controlled experimental sensor datasets, ICU EHR data are generated during routine clinical practice. Consequently, measurements are not acquired at uniform intervals. Vital signs may be recorded frequently through bedside monitoring systems, whereas laboratory investigations are ordered according to clinical necessity and may be unavailable for prolonged periods. Missing observations are represented as NaN values and are prevalent throughout the dataset. These missing values do not necessarily indicate sensor malfunction or data corruption; they may reflect normal clinical workflows, varying monitoring protocols, physician ordering practices, and heterogeneous measurement schedules across institutions. Missingness is therefore informative of real-world healthcare environments but introduces important challenges for machine learning systems.

Median imputation was employed as a robust and computationally efficient strategy to address incomplete observations while reducing the sensitivity to outliers, which are frequently encountered in critical care data. Nevertheless, any imputation strategy may obscure temporal relationships, attenuate abrupt physiological changes, and contribute to uncertainty in downstream predictions. For this reason, model performance and interpretability results are interpreted in the context of the underlying data quality, measurement sparsity, and retrospective EHR structure.

Table 1 provides a transparent overview of the main attributes and patient distribution. It distinguishes frequently sampled bedside vital signs from intermittently ordered laboratory measurements and static demographic variables. This distinction is important because the resulting heterogeneous measurement frequencies, irregular temporal sampling, and variable-dependent missingness directly shape the modeling challenge and influence the preprocessing choices. The table also summarizes the septic versus non-septic distribution used in the experimental analysis and the ICU indicators used for robustness evaluation.

### 2.3. Clinical Target Definition and Labeling Protocol

The clinical prediction target in this study is the early identification of patients who will develop sepsis prior to the recorded onset time provided in the dataset. Sepsis labels follow the annotation protocol defined in the PhysioNet/Computing in Cardiology 2019 Sepsis Challenge dataset [8]. In this dataset, sepsis onset is derived retrospectively from electronic health record (EHR) data using a definition inspired by the Sepsis-3 clinical criteria. Specifically, sepsis is identified based on the co-occurrence of suspected infection and evidence of organ dysfunction approximating a two-point increase in the Sequential Organ Failure Assessment (SOFA) score.

For each patient record, the dataset provides an estimated sepsis onset timestamp (*t_sepsis*). In accordance with the challenge formulation, the binary label SepsisLabel is shifted six hours prior to the estimated onset time. Consequently, time steps satisfying $t\geq t_{sepsis}-6$ are labeled as positive for sepsis patients, while earlier time steps are labeled as negative. All time steps for patients who do not develop sepsis remain negative.

Following this formulation, the prediction task in this study is treated as an early warning problem in which the model estimates the probability that a patient will develop sepsis within the next six hours. The annotated onset timestamp provided in the dataset serves as the temporal reference point for defining the prediction horizon and evaluating model performance. This setup enables the model to learn temporal patterns in physiological measurements and structured EHR variables that precede clinically recognized sepsis onset.

The model outputs a probability score representing the estimated risk of impending sepsis based on the observed physiological variables. In a potential clinical deployment scenario, this probability could be mapped to an alert threshold selected according to the desired trade-off between sensitivity and the false alarm rate. Lower thresholds may prioritize early detection with higher sensitivity, whereas higher thresholds may reduce false positives to mitigate alert fatigue.

Although the present study focuses on predictive modeling and a comparative evaluation of machine learning architectures, the probabilistic outputs produced by the models are compatible with integration into clinical decision support systems. In such a setting, the model would operate continuously on streaming patient data and generate alerts when the predicted risk exceeds a predefined operating threshold determined through clinical validation and institutional policy.

### 2.4. Data Preprocessing and Feature Extraction

The dataset consists of structured clinical variables recorded at hourly intervals, including vital signs, laboratory measurements, and demographic information. Prior to model training, the data are examined for missing values and inconsistencies, which are common in real-world ICU EHR data. As summarized in Table 1, the dataset combines dense bedside measurements with sparse laboratory tests and static demographic variables. Therefore, preprocessing must address not only numerical scaling but also the heterogeneous measurement frequency, irregular temporal sampling, and variable-specific missingness patterns.

Missing measurements in time-series variables are handled using forward filling to preserve the temporal continuity of clinical observations. For variables without prior observations, remaining missing values are imputed using the median calculated from the training data. Static variables, such as demographic features, are also imputed using median statistics to reduce the influence of outliers while maintaining representative population values. Median imputation was selected because ICU measurements are irregularly sampled and physiological variables often exhibit skewed distributions and extreme outliers. Median statistics are therefore more robust than mean values and avoid introducing artificial temporal trends that could occur with the aggressive interpolation of sparse laboratory variables. All median values are estimated from the training split only and then applied to validation or test data to prevent information leakage.

The missingness pattern is not uniform across feature groups. Vital signs such as heart rate, oxygen saturation, blood pressure, and respiratory rate are typically recorded more frequently, whereas laboratory and arterial blood gas variables may be absent for long intervals when tests are not clinically indicated. Consequently, missingness partly reflects ICU care processes and patient acuity rather than random data loss. The present preprocessing strategy provides a reproducible baseline for comparative modeling, while more advanced missingness-aware approaches remain an important direction for future work.

All numerical features are standardized to zero mean and unit variance using statistics computed from the training set. Standardization prevents variables with larger magnitudes from dominating the optimization process and improves the numerical stability during gradient-based learning.

Patient trajectories are organized into fixed-length temporal sequences with a one-hour resolution. To ensure consistent model inputs, sequences are padded or truncated to a predefined length. Overlapping windows with 50% overlap are generated to capture temporal dynamics while increasing the number of training samples and preserving continuity in clinical trends.

For each temporal window, labels are assigned based on the annotated sepsis onset timestamp. Specifically, a window is labeled as positive if sepsis onset occurs within the subsequent 6 h prediction horizon and negative otherwise. This formulation aligns with the early prediction objective and ensures that the model learns patterns preceding sepsis onset rather than detecting it after occurrence.

Feature extraction is performed automatically by the deep learning architectures. Convolutional layers capture short-term temporal patterns in the multivariate clinical measurements, while recurrent and attention-based components model longer-range temporal dependencies. This hierarchical representation enables the models to learn both local fluctuations and broader temporal trends associated with early sepsis development.

To address the strong class imbalance between sepsis and non-sepsis observations, class weighting is applied during training to ensure that the minority class contributes adequately to the loss function. In the experimental splits, the approximate class distribution was 85% non-sepsis and 15% sepsis. Class weights were computed from the training data in each fold using the balanced weighting rule $w_{c}=N/\left(2N_{c}\right)$, where *N* is the total number of training samples and $N_{c}$ is the number of samples in class *c*. This yields approximate weights of $w_{\mathrm{nonsepsis}}=0.59$ and $w_{\mathrm{sepsis}}=3.33$, corresponding to an effective minority-to-majority penalty ratio of about 5.7:1.

All preprocessing steps, including imputation, normalization, and sequence construction, are performed using statistics computed exclusively from the training data within each validation fold. These parameters are then applied to the corresponding test data to prevent information leakage and ensure a fair evaluation of model generalization.

Although alternative strategies such as multiple imputation or model-based handling of missingness may offer advantages, a full sensitivity analysis is left for future work.

## 3. Proposed Hybrid Framework

### 3.1. Overview of the Framework

The proposed framework is designed to model multivariate clinical time-series data derived from structured electronic health record (EHR) variables collected in intensive care units (ICUs). The input data consist of hourly measurements, including vital signs, laboratory measurements, and demographic information.

Let $X\in R^{T\times F}$ denote the patient time-series input, where *T* represents the number of temporal observations and *F* denotes the number of clinical variables. Each row of *X* corresponds to measurements recorded at a given hour, while each column represents a specific clinical variable.

The prediction task is formulated as a binary classification problem that estimates the probability that a patient will develop sepsis within a 6 h prediction horizon prior to the annotated onset timestamp. Formally, the prediction function is defined as

(1) $$y=f_{\theta }\left(X\right),$$

where $f_{\theta }$ denotes the hybrid deep learning model parameterized by θ, and y∈[0,1] represents the predicted probability of impending sepsis within the defined prediction window.

The framework integrates multiple neural network components to capture complementary temporal characteristics of ICU patient trajectories. Convolutional Neural Networks (CNNs) are employed to extract local temporal patterns and abrupt variations from multivariate physiological measurements. Recurrent architectures, including Long Short-Term Memory (LSTM) and Gated Recurrent Unit (GRU) networks, model sequential dependencies and temporal evolution in patient trajectories. Temporal Convolutional Networks (TCNs) capture long-range temporal relationships using dilated causal convolutions, enabling the efficient representation of extended temporal contexts. Transformer-based architectures leverage attention mechanisms to learn global dependencies and feature interactions across time steps.

Together, these architectures provide complementary capabilities for modeling both short-term fluctuations and long-term temporal dynamics, clarifying how different models contribute within the framework.

In this study, LSTM, GRU, and TCN models are evaluated both as standalone architectures and as components within hybrid configurations. This design enables a controlled comparison of their individual effectiveness as well as their contributions when integrated with convolutional or attention-based feature extractors for early sepsis prediction.

Hybrid architectures combine these modeling strategies to leverage their complementary strengths. Specifically, CNN-based modules first extract local feature representations, which are then processed by sequential or attention-based models such as LSTM or Transformer variants. In the CNN-ViT configuration, the multivariate time–feature matrix is treated as a two-dimensional representation and partitioned into patches to facilitate patch-based self-attention, enabling the joint modeling of temporal and feature-wise dependencies.

The outputs of these temporal modeling components are passed through fully connected layers to estimate a final risk score, representing the probability that a patient will develop sepsis within the next 6 h. This probability can be mapped to a predefined threshold to trigger early warning alerts in clinical monitoring systems.

Overall, the proposed framework provides a flexible architecture for modeling heterogeneous ICU EHR data by explicitly combining local feature extraction, sequential temporal modeling, and global attention mechanisms, enabling the effective and clinically meaningful early prediction of sepsis.

### 3.2. Deep Learning Architectures

To model complex temporal dependencies and heterogeneous clinical variables, multiple deep learning architectures are employed within the proposed framework. These architectures capture complementary characteristics of multivariate ICU clinical time-series data.

1. Convolutional Neural Networks (CNNs) [9,10,18,19]

CNNs are effective in extracting local temporal patterns and cross-variable interactions from multivariate clinical time-series measurements. For a sequential input $X\in R^{T\times F}$, a one-dimensional convolution operation along the temporal axis is defined as

(2) $$h_{t}=\sigma \left(\sum_{i=0}^{k-1}w_{i}\cdot x_{t+i}+b\right),$$

where $w_{i}$ denotes the convolutional kernel weights, *k* is the kernel size, *b* is a bias term, and σ(·) is a nonlinear activation function. Multiple filters allow the network to learn diverse temporal patterns associated with physiological changes.

For two-dimensional representations (e.g., time–feature matrices used in hybrid attention-based models), convolution can operate across both dimensions:

(3) $$h_{t,f}=\sigma \left(\sum_{i=0}^{k_{t}-1}\sum_{j=0}^{k_{f}-1}w_{i,j}\cdot x_{t+i,f+j}+b\right),$$

where $k_{t}$ and $k_{f}$ denote kernel sizes along the temporal and feature dimensions. This enables the joint modeling of temporal dynamics and feature correlations.

In this study, CNN modules are employed to extract local temporal patterns and cross-variable interactions from ICU time-series data. These representations serve as input features for subsequent sequential or attention-based models in hybrid architectures, enabling the enhanced modeling of complex physiological signals.

2. Recurrent Neural Networks (RNNs) [11,12]

RNNs are designed to capture sequential dependencies in time-series data. Long Short-Term Memory (LSTM) networks mitigate vanishing gradient problems using gated memory mechanisms.

At time step *t*, given input $x_{t}$ and previous hidden state $h_{t-1}$, the gates are computed as

(4) $$\begin{array}{cc} i_{t} & =\sigma (W_{i}x_{t}+U_{i}h_{t-1}+b_{i}), \end{array}$$

(5) $$\begin{array}{cc} f_{t} & =\sigma (W_{f}x_{t}+U_{f}h_{t-1}+b_{f}), \end{array}$$

(6) $$\begin{array}{cc} o_{t} & =\sigma (W_{o}x_{t}+U_{o}h_{t-1}+b_{o}). \end{array}$$

The candidate cell state is

(7) $${\tilde{c}}_{t}=\tanh\left(W_{c}x_{t}+U_{c}h_{t-1}+b_{c}\right),$$

and the memory update is

(8) $$c_{t}=f_{t}⊙c_{t-1}+i_{t}⊙{\tilde{c}}_{t}.$$

The hidden state is then computed as

(9) $$h_{t}=o_{t}⊙\tanh\left(c_{t}\right).$$

Gated Recurrent Units (GRUs) provide a simplified architecture with fewer parameters while maintaining the effective modeling of long-range temporal dependencies.

In this study, LSTM and GRU models are applied to ICU time-series data to capture temporal dependencies in patient trajectories. They are evaluated both as standalone architectures and as components within hybrid models, where they process feature representations extracted by convolutional layers. This design enables a systematic comparison of their effectiveness in modeling temporal dynamics and their contribution to early sepsis prediction.

3. Temporal Convolutional Networks (TCNs) [13]

TCNs employ causal and dilated convolutions to model long-range dependencies while maintaining efficient parallel computation. The dilation mechanism expands the receptive field exponentially across layers, enabling the network to capture long-term temporal trends in ICU clinical measurements.

In this study, TCN models are utilized to capture long-range temporal dependencies in ICU time-series data through dilated causal convolutions. They are evaluated as standalone architectures to assess their effectiveness in modeling extended temporal contexts compared to recurrent and attention-based models for early sepsis prediction.

4. Transformer-Based Models [12]

Transformers model global dependencies using self-attention mechanisms. For an input embedding matrix $X\in R^{T\times d}$, the scaled dot-product attention is defined as

(10) $$\mathrm{Attention}\left(Q,K,V\right)=\mathrm{softmax}\left(\frac{QK^{T}}{\sqrt{d_{k}}}\right)V,$$

where $Q=XW_{Q}$, $K=XW_{K}$, and $V=XW_{V}$.

Multi-head attention extends this mechanism by computing multiple attention operations in parallel:

(11) $$\mathrm{MultiHead}\left(X\right)=\mathrm{Concat}\left({\mathrm{head}}_{1},\ldots ,{\mathrm{head}}_{h}\right)W_{O}.$$

This architecture enables the model to learn global relationships across time steps and variables.

In this study, Transformer-based models are employed to capture global temporal dependencies and feature interactions across ICU time-series data using self-attention mechanisms. They are evaluated to assess their ability to model long-range relationships and complex variable interactions relevant to early sepsis detection.

5. Vision Transformer (ViT) [14]

ViT extends the Transformer architecture to two-dimensional inputs. The time–feature matrix is interpreted as an image $X\in R^{H\times W\times C}$, where *H* represents time steps and *W* represents clinical variables.

The input is divided into patches and projected to embeddings

(12) $$z_{0}^{i}=x_{patch}^{i}W_{E}+E_{pos}^{i}.$$

A learnable class token is added to form

(13) $$Z_{0}=\left[z_{class};z_{0}^{1};\ldots ;z_{0}^{N}\right].$$

The sequence is then processed by Transformer encoder layers consisting of multi-head attention and feed-forward networks.

In this study, ViT models are applied to time–feature representations of ICU data, where the multivariate time series is treated as a two-dimensional structure. This enables the joint modeling of temporal and feature-wise dependencies through patch-based self-attention, providing an alternative to conventional sequential modeling approaches. Specifically, each 48 h observation window is represented as a time–feature matrix after imputation and standardization. For ViT-based models, this matrix is partitioned into fixed-size non-overlapping patches, each patch is linearly projected into an embedding vector, and learnable positional encodings are added to preserve temporal and feature-order information. The resulting token sequence is processed by Transformer encoder blocks containing multi-head self-attention, feed-forward layers, residual connections, and normalization layers.

6. Hybrid Architectures

Hybrid architectures combine convolutional feature extraction with sequential or attention-based modeling. These models leverage the strengths of different neural network paradigms to capture both local and global patterns in clinical time-series data.

#### 3.2.1. CNN-LSTM

CNN layers first extract local temporal features

(14) $$f_{t}=\phi \left(W_{c}*x_{t}+b_{c}\right),$$

which are then passed to an LSTM network for the sequential modeling of temporal dependencies.

#### 3.2.2. CNN-ViT

In CNN-ViT models, convolutional layers extract low-level representations, which are converted into token embeddings for the Vision Transformer backbone. Multi-head self-attention then captures global dependencies among tokens. The CNN component is intended to capture local temporal fluctuations and short-range cross-variable patterns, whereas the ViT component models longer-range interactions across the full observation window. This design is therefore evaluated as a hybrid local–global representation strategy rather than as a clinically validated diagnostic system.

For reproducibility, the CNN-ViT implementation uses the 48 h standardized observation window as the temporal input. The CNN frontend applies one-dimensional temporal convolutions with small kernels to extract local trend features from adjacent hourly measurements. The resulting feature map is reshaped into a two-dimensional time–feature representation and segmented into non-overlapping temporal patches of 4 consecutive hours, producing 12 temporal tokens per patient window before projection. Each patch is flattened and mapped to a 64-dimensional embedding through a learnable linear projection. Learnable positional embeddings of the same dimension are added to retain the order of temporal patches, and a learnable class token is prepended to summarize the sequence for final classification.

The ViT backbone contains two Transformer encoder blocks. Each encoder block uses four self-attention heads, a feed-forward expansion dimension of 128, residual skip connections, layer normalization before the attention and feed-forward sublayers, and dropout of 0.2 after attention and dense projections. The final class-token representation is passed to a fully connected classification head with sigmoid activation to estimate the probability of sepsis within the 6 h prediction horizon. This configuration was selected to balance representational capacity with the limited size and imbalanced nature of the sepsis prediction data, avoiding an unnecessarily deep Transformer that could overfit sparse positive cases.

To avoid repeating earlier descriptions, hybrid models are summarized here as combinations of convolutional, recurrent, and attention-based modules that provide complementary temporal representations. The specific roles of each component are detailed in the model-specific paragraphs above and evaluated empirically in the ablation study.

In this study, hybrid architectures are designed to combine local feature extraction with sequential and global dependency modeling. These configurations are evaluated to determine how integrating convolutional, recurrent, and attention-based mechanisms improves the early sepsis prediction performance compared to standalone models.

### 3.3. Explainable Artificial Intelligence (XAI) Integration

To improve the interpretability and facilitate clinical adoption, the proposed framework incorporates explainable artificial intelligence (XAI) techniques at both the prediction output and model-internal levels. Because early sepsis prediction is a high-risk clinical task, model predictions must be transparent and clinically interpretable rather than functioning as opaque black-box outputs.

Three complementary XAI strategies are employed. First, Shapley Additive Explanations (SHAP) provide post hoc attribution by quantifying the contribution of each input feature to the final prediction. Second, attention-based attribution analyzes how Transformer components distribute focus across features and temporal positions during representation learning. Third, feature visualization techniques, including correlation heatmaps and principal component analysis (PCA), are used to contextualize the learned representations and verify that the model behavior aligns with the underlying clinical data structure.

By combining SHAP-based contribution analysis, attention-based focus analysis, and feature visualization, the framework provides a multi-perspective interpretation of predictions. This design supports the review of which variables and temporal regions influence model outputs, thereby improving the transparency for future AI-assisted clinical decision support research without implying clinician-validated actionability.

#### 3.3.1. SHAP-Based Feature Attribution

SHAP is employed to quantify the contributions of individual clinical variables and structured EHR features to the model’s output. For an input vector *X* and trained model $f_{\theta }\left(\cdot \right)$, the prediction can be decomposed as

(15) $$f_{\theta }\left(X\right)=\phi_{0}+\sum_{i=1}^{M}\phi_{i},$$

where $\phi_{0}$ denotes the expected model output over the background dataset, and $\phi_{i}$ represents the marginal contribution of feature $x_{i}$.

SHAP values are computed using a background reference set sampled from the training data. To ensure stability, feature attributions are averaged across validation folds and leave-one-unit-out evaluation settings. Global feature importance is obtained by computing the mean absolute SHAP value of each feature across all samples. Representative SHAP analyses are provided for multiple model families, including CNN, LSTM, GRU, Transformer, and CNN-ViT. For each architecture, the same preprocessing pipeline, background sampling strategy, and feature set were used so that the global mean absolute SHAP rankings could be compared across models. Full fold-by-fold SHAP visualizations for every experimental configuration are not included in the main manuscript to preserve readability; instead, representative multi-model SHAP summaries are provided in Supplementary Figure S2.

SHAP summary analyses consistently identify several clinical variables, such as heart rate, blood pressure, oxygen saturation, lactate, glucose, and white blood cell count, as major contributors to sepsis prediction. The directionality of the SHAP values indicates that deviations from normal physiological ranges increase the predicted sepsis risk, which aligns with established clinical knowledge of sepsis progression.

At the individual patient level, temporal SHAP trajectories illustrate how specific features gain predictive influence as sepsis onset approaches. The consistency of the SHAP rankings across validation folds suggests that the learned feature importance patterns are robust rather than artifacts of particular data partitions.

#### 3.3.2. Attention-Based Attribution Analysis

In addition to SHAP-based explanations, attention-based attribution is used to analyze how the CNN-ViT model distributes focus across features and temporal positions during representation learning. Unlike SHAP, which directly quantifies the contributions of input variables to the final prediction, attention weights reflect how information is internally weighted within Transformer layers. Therefore, attention-based attribution is interpreted as an indicator of model focus rather than a direct measure of causal importance.

##### Extraction of Attention Weights

The CNN-ViT architecture incorporates Transformer encoder blocks following the convolutional feature extractor. During inference, each Transformer block produces attention matrices of the form

(16) $$\alpha^{\left(h\right)}\in R^{T\times F},$$

where *h* indexes the attention head, *T* denotes the number of temporal steps in the input window, and *F* represents the number of feature tokens corresponding to clinical variables and structured EHR inputs.

These matrices are obtained directly from the softmax-normalized attention scores computed during the forward pass of the trained model. In practice, attention weights are extracted by enabling attention output in the Transformer modules and recording them during validation and test inference. This extraction process is performed post-training and does not modify the model predictions or training dynamics.

##### Aggregation Across Heads and Time

Because individual attention heads may specialize in distinct temporal or feature-specific patterns, attention weights are aggregated to obtain a stable summary. For a given feature *f*, the aggregated attention score is defined as

(17) $$A_{f}=\frac{1}{HT}\sum_{h=1}^{H}\sum_{t=1}^{T}\alpha_{h,t,f},$$

where *H* denotes the number of attention heads, and $\alpha_{h,t,f}$ represents the attention weight assigned to feature *f* at time step *t* by head *h*.

The resulting scores are computed for each sample, averaged across validation folds, and normalized such that

$$\sum_{f}A_{f}=1.$$

##### Mapping Attention Scores to Clinical Variables

Each attention token corresponds directly to a specific clinical variable defined in the preprocessing pipeline. This one-to-one mapping between tokens and EHR features is preserved throughout training and inference, allowing the direct interpretation of attention distributions without additional alignment procedures.

The quantitative analysis and clinical interpretation of the aggregated attention scores are presented in the Section 5, where they are examined alongside the SHAP explanations, ablation studies, and predictive performance metrics.

#### 3.3.3. Feature Visualization and Representation Analysis

To further contextualize model behavior, correlation heatmaps and principal component analysis (PCA) are employed as complementary visualization tools. Correlation heatmaps are constructed from the input feature space to examine statistical relationships among clinical variables. These visualizations reveal clinically plausible associations, such as relationships between heart rate and blood pressure or between oxygen saturation and respiratory rate, providing an overview of the dependencies present in the structured EHR data.

PCA is applied to intermediate feature embeddings extracted from the penultimate layers of the trained models. The high-dimensional representations are projected into a lower-dimensional space to visualize how patient samples are distributed according to their learned representations. The resulting projections show clearer separation between sepsis and non-sepsis samples for hybrid architectures, particularly the CNN-ViT model, compared to single-architecture baselines. This observation suggests that the hybrid models capture discriminative patterns associated with early sepsis progression.

Overall, the integration of SHAP-based feature attribution, attention-based focus analysis, and feature visualization provides a comprehensive interpretability framework. These complementary analyses offer insights into both feature-level contributions and internal representation structures, helping to bridge the gap between predictive performance and clinical interpretability in early sepsis detection.

## 4. Experimental Setup

All models are trained in a supervised learning framework using labeled outcomes of sepsis onset, enabling the networks to learn associations between structured ICU features and clinically meaningful deterioration patterns. To ensure a reliable and reproducible evaluation, the dataset is partitioned into training, validation, and testing sets using a stratified strategy that preserves the original proportions of positive and negative cases. Split ratios such as 70/10/20 or 60/20/20 allow hyperparameter tuning on unseen data while maintaining a representative held-out set for final performance assessment [40]. For smaller datasets, stratified cross-validation is employed to reduce sampling variance and stabilize performance estimates across folds.

To enhance the generalizability in the absence of true external datasets, a leave-one-unit-out cross-site validation scheme is incorporated. In this approach, data from one ICU unit (or equivalent partition) are held out as a pseudo-external test set, while the remaining units are used for training and validation. This provides a realistic estimate of model performance under slightly shifted clinical conditions. Additionally, the potential for dataset bias is acknowledged, including factors such as population skew, measurement frequency variability, and site-specific clinical practices. Clear directions for future multi-center validation are outlined to enable broader external testing once suitable datasets become available.

Given the predominance of non-sepsis cases (approximately 85%), several measures are implemented to mitigate class imbalance. Temporal sequences are padded or truncated to a uniform window of 48 time steps, and, where required by architectures such as CNN-ViT or ViT, sequences are reshaped into 2D matrices. Missing values are imputed using the median to avoid the distortion of feature distributions, and all numerical variables are standardized to zero mean and unit variance to stabilize gradient-based optimization across heterogeneous ICU features. All models are trained under the same supervised protocol using mini-batch gradient-based optimization, class-weighted binary cross-entropy, validation-based learning rate scheduling, dropout or equivalent regularization where applicable, and early stopping based on the validation performance. Hyperparameter choices, including sequence lengths, class weights, and architecture-specific reshaping, are kept consistent across folds to support a fair comparison.

Class imbalance is handled using weighted binary cross-entropy rather than resampling. For a binary label $y_{i}\in \left\{0,1\right\}$ and predicted probability ${\hat{y}}_{i}$, the training objective is

(18) $$L_{WBCE}=-\frac{1}{N}\sum_{i=1}^{N}\left[w_{1}y_{i}\log\left({\hat{y}}_{i}\right)+w_{0}\left(1-y_{i}\right)\log\left(1-{\hat{y}}_{i}\right)\right],$$

where $w_{1}$ and $w_{0}$ denote the septic and non-septic class weights, respectively. Based on the approximate 15:85 septic/non-septic distribution, the balanced weighting rule $w_{c}=N/\left(2N_{c}\right)$ gives $w_{1}\approx 3.33$ for sepsis and $w_{0}\approx 0.59$ for non-sepsis, equivalent to penalizing minority-class errors approximately 5.7 times more strongly than majority-class errors. The exact values are recomputed within each training fold to avoid leakage from validation or test partitions.

Synthetic oversampling methods such as SMOTE, time-series SMOTE, and random oversampling were considered but not adopted. The input data consist of temporally ordered ICU trajectories; generating synthetic sequences or duplicating minority windows could distort physiological progression, create unrealistic transitions between clinical states, and increase the risk of temporal information leakage when adjacent windows from the same patient are oversampled. Random undersampling was also avoided because it would discard a large proportion of non-sepsis trajectories that are needed to characterize normal ICU variability and reduce false alarms. Class-weighted optimization was therefore selected as a conservative cost-sensitive strategy that preserves the original temporal structure while reducing majority-class bias. This choice also improves the experimental transparency by making the imbalance mitigation mechanism explicit in the loss function, rather than introducing additional synthetic samples whose physiological plausibility would be difficult to verify. Despite these measures, early sepsis remains inherently challenging to detect due to subtle physiological deviations that may overlap with normal variability, contributing to residual false negatives even in well-tuned models.

Model performance is evaluated using metrics that emphasize minority-class behavior, including recall, precision, the F1-score, and the area under the precision–recall curve (AUPRC). These metrics provide a more accurate representation of clinical utility than accuracy alone, which can be inflated by the predominance of non-sepsis cases. Training stability is maintained using early stopping based on the validation loss and learning rate reduction on plateau. A batch size of 32 balances computational efficiency with stable gradient estimation, and models are trained for up to 50 epochs to ensure sufficient convergence without overfitting. Models are optimized using Adam with an initial learning rate of 0.001; the learning rate is reduced when the validation loss plateaus, and training is stopped if the validation loss fails to improve for consecutive epochs. Experiments are conducted in a Python 3.11 and JetBrains PyCharm Community Edition 2024.1 in a deep learning environment on GPU-enabled hardware when available, while all reported comparisons use the same preprocessing, split strategy, batch size, loss function, and stopping criteria to ensure that performance differences reflect architecture behavior rather than inconsistent training settings.

Hyperparameters, including convolutional kernel sizes, recurrent units, attention heads, embedding dimensions, and dropout rates, are optimized using a hybrid strategy: grid search is applied to key structural components, while random search explores broader parameter configurations. These tuning strategies improve generalization in the presence of class imbalance, temporal heterogeneity, and overlapping physiological patterns characteristic of early sepsis prediction.

### 4.1. Weighted Ensemble Framework

The weighted ensemble is included as a comparative benchmark rather than as a newly proposed model architecture. Its purpose is to test whether combining predictions from heterogeneous temporal models improves the robustness relative to the best individual model. Let *M* denote the number of trained base models and let $p_{m}\left(x_{i}\right)\in \left[0,1\right]$ be the predicted sepsis probability from model *m* for patient window $x_{i}$. The ensemble prediction is computed as a convex weighted average:

(19) $${\hat{p}}_{ens}\left(x_{i}\right)=\sum_{m=1}^{M}\alpha_{m}p_{m}\left(x_{i}\right),\;\sum_{m=1}^{M}\alpha_{m}=1,\;\alpha_{m}\geq 0.$$

The final binary decision is obtained by applying the same operating threshold used for individual models:

(20) $${\hat{y}}_{ens}\left(x_{i}\right)=I\left({\hat{p}}_{ens}\left(x_{i}\right)\geq \tau \right),$$

where τ is selected on the validation set to balance recall and precision under the early warning objective.

Ensemble weights are assigned using validation F1-scores rather than manual selection. For each training fold, the validation F1-score of model *m*, denoted $F1_{m}^{val}$, is converted into a normalized contribution weight:

(21) $$\alpha_{m}=\frac{F1_{m}^{val}}{\sum_{j=1}^{M}F1_{j}^{val}}.$$

This performance-proportional weighting gives greater influence to models with stronger minority-class detection while retaining contributions from diverse architectures. Weights are computed only from validation data within each fold and then fixed for the corresponding test evaluation to avoid test-set leakage. No additional meta-learner is trained, and no test labels are used to optimize ensemble coefficients. Therefore, the ensemble should be interpreted as a transparent probability fusion baseline, not as a novel stacked architecture or a separate methodological contribution.

### 4.2. Evaluation Metrics

Given the imbalanced nature of the dataset, multiple complementary evaluation metrics are employed to avoid misleading conclusions from accuracy alone. Accuracy measures the overall proportion of correctly classified samples but can be inflated by the majority class [41]. Precision quantifies the fraction of predicted positives that are true positives, while recall (sensitivity) captures the proportion of actual positives that are correctly identified [42]. The F1-score, defined as the harmonic mean of precision and recall, balances these two aspects and is particularly important in imbalanced clinical prediction tasks [43]. The inference time is also reported to evaluate computational efficiency and assess the feasibility of deploying models in real-time ICU settings, where timely predictions are critical for patient management [44]. Recall and the F1-score are emphasized to measure the model’s effectiveness in identifying positive sepsis cases, as missed detections can have severe clinical consequences [44].

Evaluation metrics are computed not only on the standard stratified test set but also using a leave-one-unit-out cross-site validation scheme, where data from one ICU unit are held out as a pseudo-external test set. This approach provides a more realistic assessment of model generalizability across slightly shifted clinical environments. Confusion matrices, feature distribution plots, and dimensionality reduction analyses using principal component analysis (PCA) are also applied to the cross-site held-out units to assess minority-class separability and evaluate the overall robustness of the models. These analyses consistently show that negative cases are classified with high confidence, while positive sepsis cases remain more challenging, reflecting class imbalance and overlapping physiological patterns [44,45].

Furthermore, feature-space explorations, including distribution plots, correlation heatmaps, and PCA, are performed to understand the underlying data structures and class separability [45]. PCA reveals that the minority class is not linearly separable from the majority class, explaining why even advanced deep learning models with attention mechanisms may struggle to reliably distinguish early sepsis patterns. These evaluations collectively ensure a comprehensive assessment of model performance, robustness, interpretability, and potential clinical applicability.

### 4.3. Reproducibility Considerations

To improve the experimental reproducibility, all evaluated architectures used the same preprocessing pipeline, identical cross-validation splits, fixed random seeds, and consistent evaluation metrics. Hyperparameter tuning was conducted within the same training–validation framework to minimize comparison bias between models. Implementation details, preprocessing configurations, and training settings are described to facilitate future benchmarking and replication studies.

## 5. Results

### 5.1. Baseline and Comparative Models

The proposed hybrid architectures, including CNN-LSTM and CNN-ViT, are systematically benchmarked against single-model baselines (1D-CNN, 2D-CNN, LSTM, GRU, Bi-LSTM, TCN, and Transformer) using the same structured EHR time-series data.

This comparison is designed to evaluate how different architectural choices—convolutional, recurrent, and attention-based—capture complementary temporal characteristics in ICU patient trajectories. Unlike prior work that focuses on a single model, this study provides a unified experimental framework for assessing multiple deep learning paradigms under consistent data preprocessing and evaluation settings.

The results show that hybrid and Transformer-based models provide modest but consistent improvements over simpler baselines. However, the performance differences across models remain relatively small, particularly in the F1-score and AUPRC. This indicates that the challenge of early sepsis prediction is strongly influenced by data characteristics such as class imbalance, label uncertainty, and heterogeneity across ICU stays, rather than model complexity alone.

These findings highlight that architectural improvements alone may not lead to substantial performance gains and emphasize the importance of robust evaluation, interpretability, and clinically meaningful validation in developing deployable early warning systems.

### 5.2. Predictive Performance

Table 2 summarizes the predictive performance of all evaluated models, including a weighted ensemble of base learners. Overall, single-model baselines provide competitive performance. For instance, the 1D-CNN achieves an F1-score of 0.422 and an AUPRC of 0.43, while recurrent models such as LSTM and GRU improve the performance to F1-scores of 0.445 and 0.443, respectively. Transformer-based models further enhance the performance, achieving an F1-score of 0.452 and an AUPRC of 0.47, reflecting their ability to capture long-range temporal dependencies in clinical time-series data.

Hybrid architectures, including CNN-LSTM and CNN-ViT, yield consistent but incremental improvements. Among all models, CNN-ViT achieves the highest performance with an F1-score of 0.454 and an AUPRC of 0.48. However, the margin of improvement over the Transformer (F1-score of 0.452) and the weighted ensemble (F1-score of 0.453) remains small, indicating that no single architecture provides a decisive advantage for this task. In particular, the accuracy difference between CNN-ViT (0.8825) and the weighted ensemble (0.8805) is only 0.0020, and their confidence intervals overlap substantially. Therefore, this difference should not be interpreted as statistically significant or clinically meaningful on its own.

The weighted ensemble achieves performance comparable to that of the best individual model, suggesting that combining diverse model predictions can enhance robustness, although it does not substantially outperform well-designed single architectures.

Overall, the results indicate that the performance differences across modern deep learning architectures are modest for early sepsis prediction. While hybrid models consistently provide slight improvements, the gains are incremental rather than transformative. These findings also highlight the importance of using evaluation metrics such as the F1-score and AUPRC in imbalanced clinical datasets, where accuracy alone may overestimate model effectiveness. More importantly, the results suggest that the primary limitations are associated with data characteristics, such as class imbalance, label uncertainty, and patient heterogeneity, rather than model capacity alone.

These observations underscore the need for future work to focus on improved label definition, enhanced data quality, and clinically grounded validation strategies, rather than relying solely on increasing architectural complexity.

### 5.3. Statistical Comparison of Model Performance

To assess whether the observed performance differences are statistically meaningful, paired statistical testing was performed across stratified cross-validation folds using the F1-score and AUPRC as the primary evaluation metrics. Because performance distributions across folds may not satisfy normality assumptions, the non-parametric Wilcoxon signed-rank test was used for pairwise model comparison. Comparisons between CNN-ViT and simpler baseline architectures, including the 1D-CNN and 2D-CNN, showed consistent improvements across folds. However, comparisons between CNN-ViT and the weighted average ensemble yielded only small numerical differences, with statistical significance not consistently observed across evaluation metrics (p>0.05). These findings reinforce that the performance advantage of CNN-ViT over strong hybrid or ensemble approaches is modest rather than decisive. Accordingly, the results should be interpreted as evidence that hybrid architectures provide stable and practically meaningful improvements for minority-class detection, while recognizing that several advanced temporal architectures achieve broadly comparable performance under the present experimental conditions.

### 5.4. Confidence Interval Analysis

To quantify variability across validation folds, 95% confidence intervals were estimated for recall, the F1-score, and the AUPRC using fold-level variability estimates and bootstrap-style resampling of cross-validation predictions. As can be seen in Table 3 the resulting intervals indicate moderate overlap among the strongest-performing models, particularly CNN-ViT, Transformer, TCN, and the weighted average ensemble. Although CNN-ViT achieved the highest average performance across most metrics, the overlapping confidence intervals further support the interpretation that the observed gains are incremental rather than definitive evidence of architectural superiority.

### 5.5. Statistical Interpretation of Performance Differences

Because the dataset is strongly imbalanced, accuracy is reported only as a secondary descriptive metric. A model can obtain high accuracy by correctly classifying the majority non-sepsis class while still missing clinically important sepsis cases. Therefore, the interpretation of model utility in this study prioritizes recall, the F1-score, and the AUPRC, which more directly reflect minority-class detection and the precision–recall trade-off relevant to early warning systems.

The confidence intervals and paired comparisons in Table 2 are intended to provide a transparent indication of variability across validation folds rather than definitive evidence of statistical superiority. The intervals are relatively narrow because they are computed from repeated fold-level estimates under a fixed preprocessing and evaluation protocol, and they do not replace a full patient-level non-parametric bootstrap analysis. Moreover, several top-performing models have overlapping confidence intervals and very small differences in F1-score and AUPRC. Consequently, the apparent ranking among CNN-ViT, Transformer, CNN-LSTM, TCN, and the weighted ensemble should be interpreted cautiously. The results support the conclusion that hybrid and sequence-aware models are competitive and clinically promising, but they do not establish a large or statistically decisive advantage of one architecture over all alternatives.

The reported accuracies of CNN-ViT (0.8825) and the weighted ensemble (0.8805) are extremely close. The absolute difference of 0.0020 corresponds to only 0.20 percentage points and is not sufficient to claim statistically significant superiority, especially given the overlapping 95% confidence intervals of 0.8755–0.8895 and 0.8735–0.8875. Therefore, CNN-ViT should not be interpreted as decisively better than the ensemble on the basis of the accuracy alone. Instead, CNN-ViT, Transformer, TCN, CNN-LSTM, Bi-LSTM, and the weighted ensemble are treated as a top-performing group with broadly comparable predictive behavior.

The primary interpretation is therefore shifted from ranking models by marginal accuracy differences toward comparing modeling behavior under class imbalance. Recall, the F1-score, and the AUPRC are emphasized because they better reflect the ability to detect the minority sepsis class. The main contribution is the controlled comparison of temporal modeling strategies, computational trade-offs, and explanation consistency, rather than a claim that one architecture achieves overwhelming or statistically decisive superiority.

### 5.6. Observations and Rationale

The comparative evaluation highlights the role of hybrid architectures in handling heterogeneous ICU data. Models such as CNN-LSTM and CNN-ViT demonstrate consistent, albeit modest, improvements over simpler baselines, including 1D-CNN, 2D-CNN, and standalone recurrent models. These architectures combine convolutional layers for local feature extraction with sequential or attention-based components for the modeling of temporal dynamics. For example, CNN-LSTM achieved accuracy of 0.8745, recall of 0.7400, an F1-score of 0.449, and an AUPRC of 0.46, while CNN-ViT reached 0.8825 accuracy, 0.7480 recall, an F1-score of 0.454, and an AUPRC of 0.48. Although these results suggest that integrating local and global representations is beneficial, the magnitude of improvement remains limited.

Transformer and TCN models also demonstrate strong capabilities in capturing long-range temporal dependencies. The Transformer achieves an F1-score of 0.452 and AUPRC of 0.47, while the TCN attains an F1-score of 0.451 and AUPRC of 0.46, indicating performance comparable to hybrid models. Similarly, Bi-LSTM improves the recall to 0.7380 and F1-score to 0.450, highlighting the value of bidirectional temporal modeling. These findings suggest that multiple architectural paradigms can achieve similar levels of performance when applied to this task.

To further assess whether hybrid architectures provide advantages beyond simple model combination, a weighted average ensemble of base models was evaluated, with weights proportional to the individual F1-scores. The ensemble achieved an F1-score of 0.453 and AUPRC of 0.475, which are comparable to those of the best-performing individual model (CNN-ViT). This indicates that, while hybrid architectures offer a structured way to combine modeling capabilities, their performance gains over well-constructed ensembles are incremental. Accordingly, the CNN-ViT and weighted ensemble results should be viewed as practically similar rather than as evidence of a statistically significant difference.

Despite these improvements, performance remains constrained by the rarity and clinical ambiguity of sepsis events. High recall often coincides with lower precision, as observed in the 1D-CNN baseline. The F1-score and AUPRC better summarize the sensitivity–precision trade-off than accuracy alone in this imbalanced prediction setting.

Overall, these observations indicate that, while hybrid and sequence-aware architectures provide consistent improvements, the gains are modest and not decisive. This suggests that future progress in early sepsis prediction may depend more on improvements in data quality, label definition, and clinically informed modeling strategies than on architectural complexity alone. In particular, the 88% accuracy of CNN-ViT should not be interpreted as primary evidence of clinical usefulness; the more relevant findings are its recall of 0.7480 and AUPRC of 0.48, which better reflect its performance under severe class imbalance.

### 5.7. Interpretability Analysis

**Figure 2:**
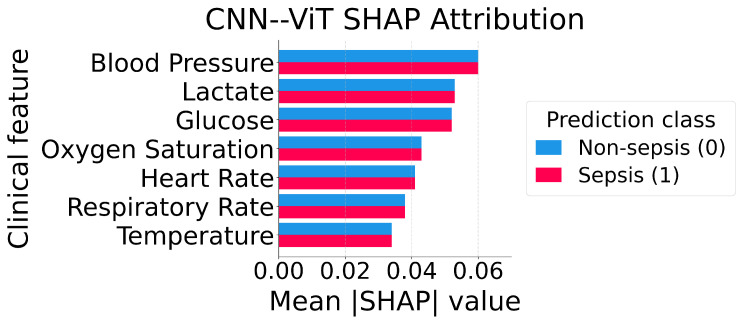
SHAP summary plot for CNN-ViT showing output-level feature importance. The legend uses clinically explicit labels, “Non-sepsis class (0)” and “Sepsis class (1)”, rather than numeric class names alone. The *x*-axis reports mean absolute SHAP values, i.e., average absolute contributions to the model output, and the *y*-axis lists the clinical features. Longer horizontal bars indicate a stronger average influence of a feature on the corresponding class prediction. Vital signs and laboratory variables contribute to predictive risk; these SHAP values should be interpreted as post hoc feature attributions rather than causal clinical effects. The legend, axis labels, and annotation distinguish class labels, clinical features, and SHAP contribution magnitudes. The plot therefore supports physiological plausibility assessment but does not establish that changing a variable would causally alter sepsis risk.

Explainable AI (XAI) methods, including SHAP and attention visualization, provide insight into the learned representations for both hybrid models and the weighted ensemble. The SHAP summary plots (Figure 2) consistently highlight vital signs such as blood pressure, lactate, glucose, oxygen saturation, and heart rate as influential predictors. Both high and low deviations in these variables appear informative for sepsis prediction, which is broadly consistent with clinical expectations. The visualization separates three concepts: the prediction class shown in the legend, the clinical feature listed on the y-axis, and the mean absolute SHAP contribution shown on the x-axis. Thus, the blue and red bars identify non-sepsis and sepsis class attributions, respectively, while the bar length represents the average magnitude of feature influence rather than the raw clinical measurement value.

Supplementary Figure S2 extends the SHAP analysis to representative CNN, LSTM, GRU, Transformer, and CNN-ViT models. Across these architectures, MAP, lactate, heart rate, oxygen saturation, WBC, and glucose remain among the most influential variables, suggesting that the models learn broadly consistent physiological risk patterns. CNN-based models tend to assign relatively higher importance to densely sampled vital signs and local fluctuations, whereas recurrent and Transformer-based models distribute importance more strongly across temporally evolving laboratory and hemodynamic variables. These differences are expected because the architectures encode temporal information differently; nevertheless, the overlap among the top-ranked features supports the robustness of the overall XAI interpretation.

To assess the robustness, SHAP analyses were conducted across multiple stratified folds and leave-one-unit-out splits. The resulting feature rankings remained largely stable across different data partitions, suggesting that the models capture consistent patterns rather than dataset-specific artifacts. Temporal SHAP and attention visualizations show general alignment between sequential feature contributions and attention weights, highlighting time windows preceding sepsis onset that contribute most strongly to predictions. Case-level attention trajectories further illustrate how the predicted risk evolves over time for individual patients.

The features identified by XAI methods, including the mean arterial pressure (MAP), lactate, white blood cell count (WBC), heart rate, and oxygen saturation, correspond to clinically recognized indicators associated with sepsis. This alignment suggests that the models rely, at least in part, on physiologically plausible signals rather than purely spurious correlations.

Attention maps from the Transformer component of the CNN-ViT model indicate temporal regions that contribute more strongly to the prediction, particularly during periods of physiological instability. The weighted ensemble exhibits broadly similar feature importance patterns when analyzed using post hoc attribution methods, although it appears less sensitive to localized temporal variations. This observation suggests that hybrid architectures may provide a more structured way to integrate local and global temporal information, although the differences remain subtle. Attention visualizations are internal model focus summaries; they should be interpreted together with SHAP, the ablation results, and clinical plausibility rather than as standalone expert clinical explanations.

#### 5.7.1. Attention-Based Attribution Results

In addition to SHAP-based explanations, attention-based attribution provides a complementary perspective on how the Transformer components of the CNN-ViT model allocate focus across physiological features and time during sequence modeling. Aggregated attention scores indicate that core vital signs tend to receive higher emphasis, suggesting their importance in forming global temporal representations.

Table 4 reports the normalized attention-based attribution scores obtained from the CNN-ViT model. Heart rate receives the highest attention weight, followed by the mean arterial pressure (MAP), oxygen saturation (SpO_2_), and lactate. These features are widely recognized as clinically relevant indicators associated with sepsis. While attention weights do not provide a direct causal explanation, their alignment with established physiological markers suggests that the model focuses on meaningful signals rather than purely spurious patterns. Respiratory rate and temperature provide additional contextual information but receive comparatively lower attention.

Temporal inspection of the attention maps further highlights time windows preceding sepsis onset during which the attention values increase in response to physiological deviations. These patterns provide insight into when the model places greater focus during prediction. However, such observations should be interpreted as indicative rather than definitive explanations of model behavior.

#### 5.7.2. SHAP–Attention Attribution Comparison

Because attention weights do not necessarily constitute faithful explanations, we explicitly compared the SHAP-derived feature importance with attention-based attribution. SHAP estimates how strongly each input variable contributes to the final prediction output, whereas attention weights indicate how the Transformer component distributes representational focus across feature tokens and time steps during sequence modeling. Therefore, agreement between the two methods strengthens interpretability, but disagreement should not be interpreted as model failure.

The two approaches showed the strongest agreement for heart rate, mean arterial pressure, oxygen saturation, and lactate, which were consistently emphasized by both SHAP and attention attribution. These variables are clinically plausible sepsis indicators and suggest that the model relies on physiologically meaningful patterns. Partial disagreement was observed for variables such as glucose, white blood cell count, respiratory rate, and temperature. For example, SHAP assigned relatively high importance to laboratory and metabolic variables that directly affected the predicted risk score, whereas attention attribution sometimes assigned greater weight to frequently sampled vital signs because they provide dense temporal context for sequence representation. Conversely, respiratory rate and temperature occasionally received moderate attention despite lower SHAP rankings, suggesting that they may help to contextualize temporal deterioration without independently dominating the final prediction.

These differences highlight the complementary nature of the two XAI approaches. SHAP is better interpreted as output-level feature contributions, while attention is better interpreted as having an internal temporal and contextual emphasis. Accordingly, attention does not always equal explanation, and attention maps should not be treated as causal evidence. In this study, the attention-based results are used as supportive evidence alongside the SHAP, ablation analysis, and cross-unit stability results, rather than as a standalone explanation of clinical causality.

##### Illustrative Computation Example

To clarify the aggregation process defined in (17), we provide an illustrative example. Consider a simplified setting with H=4 attention heads and T=48 temporal steps, and assume that heart rate corresponds to feature token f=1.

For a single attention head, the temporal mean attention assigned to heart rate is computed as

$$\frac{1}{T}\sum_{t=1}^{T}\alpha_{h,t,1}=0.20.$$

Averaging attention score

$${\bar{A}}_{\mathrm{HR}}=\frac{1}{H}\sum_{h=1}^{H}\left(\frac{1}{T}\sum_{t=1}^{T}\alpha_{h,t,1}\right)=0.205.$$

This computation is performed for each physiological feature, producing a set of unnormalized scores $\left\{{\bar{A}}_{f}\right\}$. These values are then averaged across all samples and validation folds and normalized as

(22) $$A_{f}^{\mathrm{norm}}=\frac{{\bar{A}}_{f}}{\sum_{f^{\prime }}{\bar{A}}_{f^{\prime }}}.$$

After normalization across all features, the relative importance of heart rate corresponds to the value reported in Table 4 ($A_{\mathrm{HR}}^{\mathrm{norm}}=0.214$). The numerical values in this example are illustrative; the table reports statistics aggregated over the full dataset and cross-validation folds.

##### Mapping Attention Scores to Physiological Features

Each attention token corresponds to a predefined physiological variable or structured EHR feature as specified in the input representation. This one-to-one mapping is fixed during preprocessing and preserved throughout model training and inference. Consequently, aggregated attention scores can be associated with clinically interpretable variables (e.g., heart rate, MAP, SpO_2_, lactate), although such associations should be interpreted as model-derived signals rather than definitive clinical importance.

### 5.8. Ablation Studies

**Figure 3:**
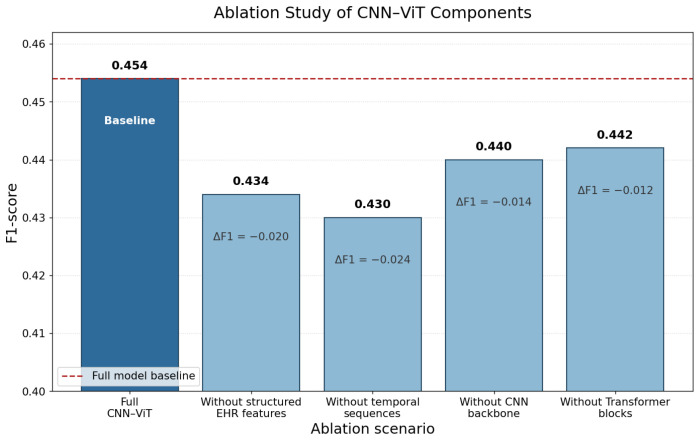
Ablation study results for CNN-ViT. Bars indicate F1-score under each ablation scenario, illustrating the contribution of structured EHR features, temporal sequences, CNN backbone, and Transformer blocks. The analysis highlights that each component significantly contributes to minority-class detection and overall predictive performance.

The ablation experiments quantify the contribution of each component in the CNN-ViT architecture (Table 5, Figure 3). The full model achieves an F1-score of 0.454, consistent with the predictive performance results, serving as the benchmark. To quantify the impact of removing each component, the change in F1-score is defined as

(23) $$\begin{array}{c} \Delta F1={F1}_{\mathrm{scenario}}-{F1}_{\mathrm{full}\;\mathrm{model}}, \end{array}$$

where ${F1}_{\mathrm{scenario}}$ is the F1-score after removing a specific component, and ${F1}_{\mathrm{full}\;\mathrm{model}}$ is the F1-score of the complete CNN-ViT model. A negative ΔF1 indicates a performance drop relative to the full model, confirming the importance of the removed component.

Figure 3 visualizes these differences. Removing structured EHR features reduces the F1-score to 0.434 (ΔF1 = −0.020), demonstrating the predictive value of combining tabular clinical data with physiological signals. Excluding temporal sequences results in the steepest decline (F1 = 0.430, ΔF1 = −0.024), highlighting the necessity of modeling longitudinal patient dynamics. Ablating the CNN backbone lowers the F1-score to 0.440 (ΔF1 = −0.014), reflecting the importance of local feature extraction, while removing the Transformer blocks leads to a slight drop (F1 = 0.442, ΔF1 = −0.012), emphasizing their role in global dependencies.

These results collectively demonstrate that CNN-ViT’s strength arises from integrating multiple structured EHR variable groups, combining local and global representations, and leveraging both sequential and tabular information. Ablating any single component consistently weakens the predictive performance, reaffirming the necessity of hybrid heterogeneous–structured data designs for robust clinical decision support, particularly in detecting the minority sepsis class.

### 5.9. XAI Consistency Across Folds and Units

To strengthen the interpretability, we quantified the stability of the SHAP feature importance and temporal attention patterns across stratified cross-validation folds and leave-one-unit-out splits. Table 6 reports the Spearman rank correlation of the top-10 SHAP features and the normalized temporal attention alignment for each model. The high correlation values indicate that the explanation patterns remain stable despite differences in training distribution or when an entire ICU unit is held out.

Across both folds and leave-one-unit-out splits, the CNN-ViT hybrid model shows the strongest SHAP consistency (0.91 ± 0.03) and attention alignment (0.88 ± 0.04), suggesting that its reasoning process remains stable even under substantial distributional shifts. The CNN-LSTM and weighted ensemble models exhibit similarly high but slightly lower consistency, reflecting robust generalization across heterogeneous ICU units.

These results confirm that the models are not relying on fold-specific or unit-specific artifacts. Instead, they repeatedly prioritize physiologically meaningful predictors, such as the mean arterial pressure, lactate, and white blood cell count, and focus attention on similar pre-onset temporal segments. This quantitative evidence provides a stronger demonstration of the explanation stability than isolated case visualizations and supports the robustness of the hybrid and ensemble architectures in retrospective ICU benchmark scenarios.

### 5.10. Data and Feature Visualization

To better understand the dataset characteristics and model behavior, we conducted visual analyses spanning class distributions, temporal patterns, and feature variability. These visualizations provide complementary insights into the challenges of sepsis prediction and the strengths of the learned representations.

#### 5.10.1. Class Distribution and Representative Sequences

**Figure 4:**
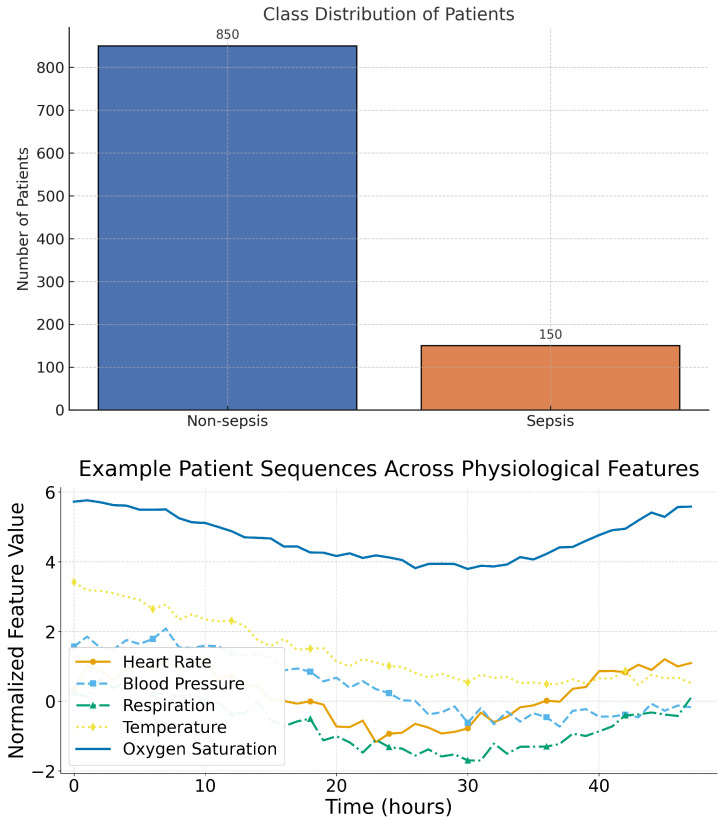
Class distribution and example patient sequences. The top panel shows the substantial class imbalance between sepsis (minority) and non-sepsis patients. The bottom panel shows normalized temporal trajectories for multiple physiological variables, illustrating intra-patient variability and inter-patient heterogeneity in ICU dynamics. Temporal patterns identified by attention and SHAP align with critical sepsis indicators.

The top panel of Figure 4 highlights the marked imbalance, with 850 non-sepsis versus 150 sepsis cases. Such disproportion significantly influences model performance, particularly the precision–recall behavior, as detecting the minority class remains inherently more challenging.

The bottom panel presents normalized sequences for a representative patient across multiple physiological variables, including heart rate, blood pressure, respiration, temperature, and oxygen saturation. These trajectories reveal notable intra-patient variability, as each vital sign follows a distinct temporal pattern. At a broader level, they reflect inter-patient variability, since the timing and progression of physiological deterioration differ across the cohort. Together, these panels underscore the dual challenges of class imbalance and heterogeneous temporal dynamics, reinforcing the importance of robust preprocessing, normalization, and temporal modeling strategies to retain clinically relevant patterns while mitigating noise.

#### 5.10.2. Low-Dimensional Feature Projections

PCA was included as an exploratory visualization tool rather than as a primary predictive or statistical analysis. Its purpose is to provide a low-dimensional view of whether raw structured ICU features and learned CNN-ViT embeddings exhibit visually different class organization. The projection uses the first two principal components, which summarize the largest linear sources of variance in the represented feature space. Because PCA is linear and unsupervised, the components are not optimized for sepsis discrimination and may not preserve nonlinear temporal relationships that are central to deep sequence models.

**Figure 5:**
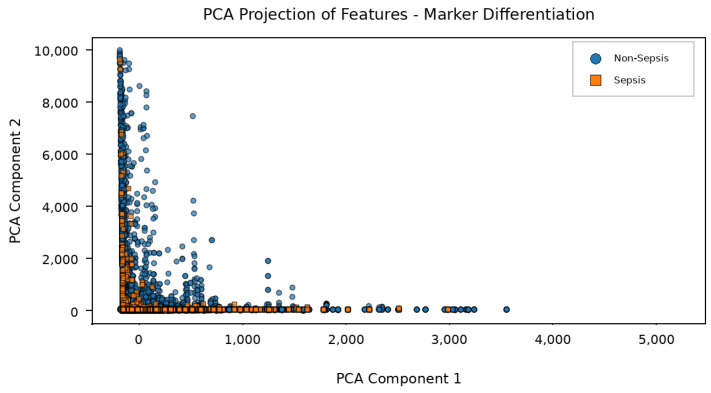
PCA projection of raw patient features. The visualization uses consistent class colors, clear axis labels, and an aligned plotting style. Some clustering of sepsis versus non-sepsis patients is observed, although significant overlap reflects the ambiguity of raw clinical signals. Because PCA is an unsupervised linear projection, this figure should be interpreted as a qualitative visualization of the feature-space structure rather than as a classifier performance result.

To examine the global structure of the raw data, Figure 5 shows patient features projected into two dimensions using PCA. The projection reveals the partial clustering of sepsis and non-sepsis cases, but substantial overlap remains, reflecting the inherent difficulty of discriminating sepsis onset from raw clinical measurements alone. This ambiguity is clinically plausible: early sepsis symptoms often resemble normal physiological variability, resulting in overlapping patterns in the input space. Despite this, the modest separation suggests the presence of global trends that deep models can exploit, enhancing their ability to extract predictive signals from complex, high-dimensional data. This observation should be interpreted cautiously: apparent proximity or separation in the PCA plane does not by itself establish predictive separability, because information outside the first two components and nonlinear temporal interactions may be omitted.

#### 5.10.3. Feature-Space Separability

**Figure 6:**
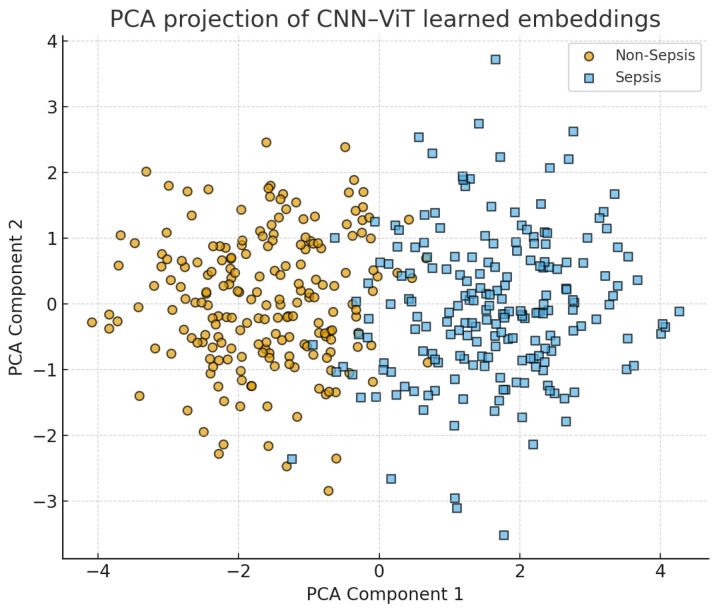
PCA projection of CNN-ViT learned embeddings. The figure applies the same class colors, marker style, and labeling convention as the raw-feature PCA plot, enabling direct visual comparison. Compared to raw features, clearer class separation is evident, although overlapping boundary regions highlight persistent prediction challenges. Residual overlap emphasizes that the learned embedding improves separability but does not eliminate uncertainty in early sepsis prediction.

To assess the structure learned by the models beyond raw inputs, Figure 6 applies PCA to latent embeddings extracted from the penultimate layer of the CNN-ViT hybrid. Compared to the raw features, the embedding space exhibits much clearer separation between sepsis and non-sepsis cases, indicating that the architecture effectively captures discriminative temporal and structured patterns. Nevertheless, residual overlap persists near class boundaries, highlighting patients whose physiological trajectories remain inherently ambiguous. This explains why the precision does not reach ideal levels: the model amplifies subtle but informative signals, yet uncertainty in early sepsis detection cannot be fully eliminated. These findings align with the ablation study, where the combination of structured EHR inputs, temporal sequences, CNN local feature extraction, and Transformer global modeling contributed to consistent improvements in recall and AUPRC. The PCA embedding plot is therefore used as supportive qualitative evidence that the trained representation is more organized than the raw input space, not as independent validation of model accuracy or clinical separability. Collectively, these visualizations demonstrate both the progress achieved and the persistent challenges in early sepsis modeling.

#### 5.10.4. Training Dynamics

**Figure 7:**
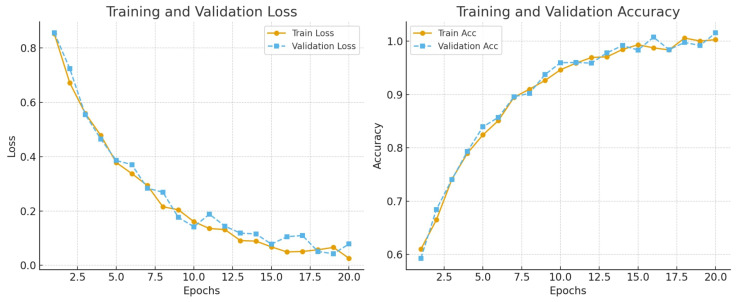
Training and validation loss (**left**) and accuracy (**right**) for CNN-ViT. Learning rate scheduling and early stopping ensured smooth convergence and effective generalization.

Figure 7 presents the training and validation curves for CNN-ViT. Both the loss and accuracy plots indicate stable convergence, facilitated by learning rate scheduling and early stopping to prevent overfitting. The close alignment of the training and validation trajectories further confirms the good generalization, supporting the robustness of the hybrid architecture. Training–validation comparisons for all evaluated architectures are provided in Supplementary Figure S1. These supplementary plots include the training loss, validation loss, training accuracy, and validation accuracy trends for the CNN, LSTM, GRU, TCN, Transformer, CNN-ViT, and weighted ensemble benchmark. Because the weighted ensemble does not introduce independently trainable neural parameters, its supplementary panel should be interpreted as a validation-level probability fusion stability curve rather than as a separately optimized model loss.

Across architectures, the training and validation curves show broadly stable convergence without large divergence between the training and validation accuracy or a sustained increase in validation loss after the training loss decreases. This pattern suggests that the models learned generalizable temporal representations rather than simply memorizing the training data. The CNN and recurrent models converge rapidly but plateau at slightly lower validation performance, whereas the TCN and Transformer-based models converge more gradually, reflecting their larger temporal modeling capacities. CNN-ViT exhibits the smoothest convergence among the individual models, consistent with its comparatively strong recall, F1-score, and AUPRC. Importantly, the validation curves remain close to the corresponding training curves, and no architecture shows the typical overfitting signature of continuously improving training performance accompanied by deteriorating validation performance. These observations support the conclusion that the reported performance differences are not caused by severe overfitting in a single model, but rather reflect differences in temporal representation learning, optimization stability, and model capacity.

#### 5.10.5. Confusion Matrices and Misclassification Patterns

**Figure 8:**
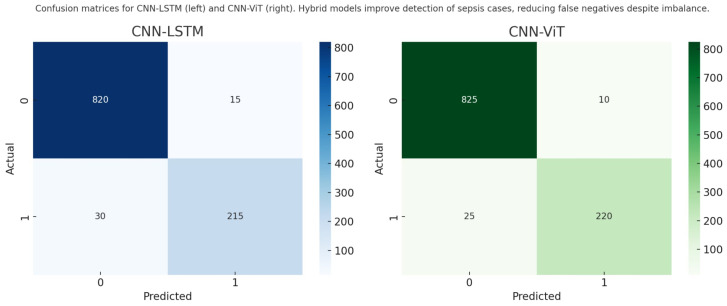
Confusion matrices for CNN-LSTM (**left**) and CNN-ViT (**right**).

Figure 8 compares the confusion matrices for CNN-LSTM, CNN-ViT, and the weighted average ensemble. Both hybrid models and the ensemble substantially reduce false negatives relative to the simpler baselines, which is crucial in clinical practice, where failing to detect sepsis can have severe consequences. The weighted average ensemble slightly improves the overall F1-score and balances false positives and false negatives, demonstrating that combining complementary predictions from multiple models can further enhance minority-class detection. The remaining false positives reflect the combined effects of class imbalance and partial feature overlap. These patterns highlight the trade-off between sensitivity and specificity and underscore the continued importance of strategies such as class weighting or cost-sensitive learning.

#### 5.10.6. Discriminative Ability

**Figure 9:**
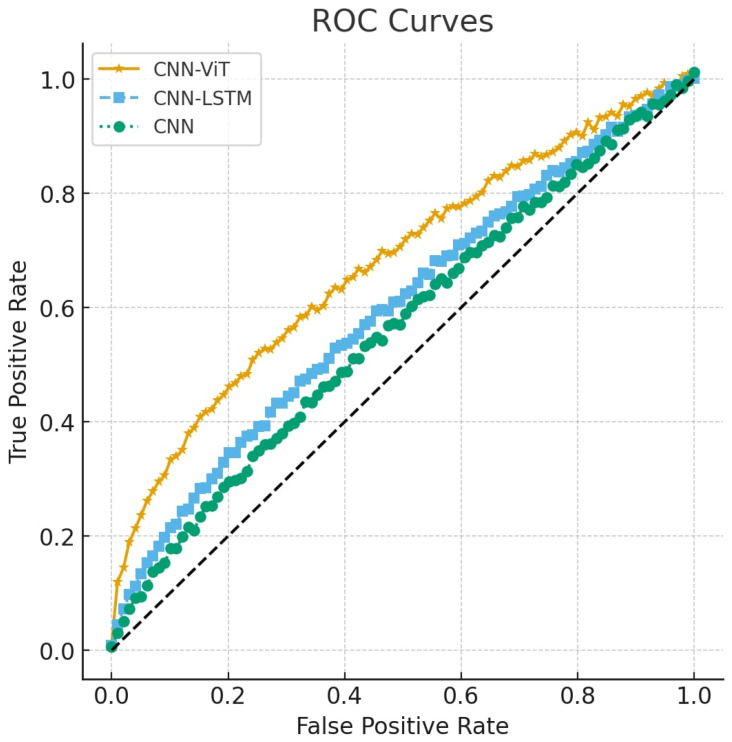
ROC curves for CNN-LSTM, CNN-ViT, and weighted average ensemble. The plot uses consistent line styles, legends, and axis labeling for clarity. Ensemble provides improved discriminative performance across thresholds. The black dotted diagonal line represents the no-skill baseline (random classifier, AUC = 0.5), used as a reference to indicate performance above chance level. Because ROC curves can appear optimistic under class imbalance, these results are interpreted alongside the AUPRC and recall rather than in isolation.

Figure 9 presents the receiver operating characteristic (ROC) curves for CNN-LSTM, CNN-ViT, and the weighted average ensemble. The ROC illustrates the true positive rate (sensitivity) against the false positive rate across thresholds. CNN-ViT maintains the highest AUC among individual models, while the ensemble slightly improves both the ROC and precision–recall metrics, reflecting better trade-offs between sensitivity and specificity.

#### 5.10.7. Performance Versus Efficiency Trade-Offs

**Figure 10:**
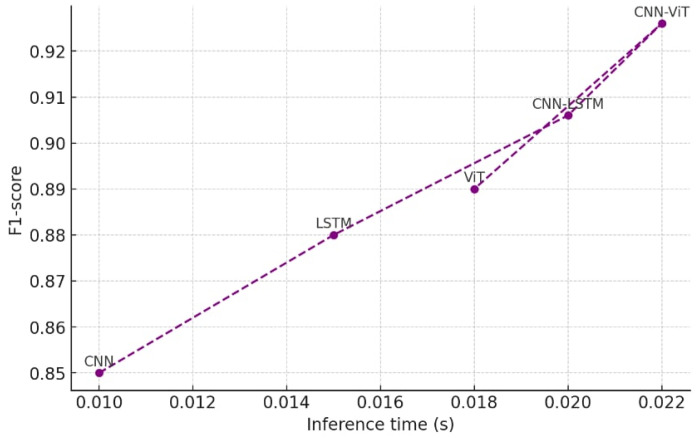
F1-score versus inference time for CNN-LSTM, CNN-ViT, and weighted average ensemble. Ensemble slightly improves F1 but requires higher inference time.

Figure 10 illustrates the F1-score versus inference time for all models, including the ensemble. CNN-LSTM and CNN-ViT provide high F1-scores with efficient inference, while the ensemble slightly increases the F1 at the cost of additional computation. This demonstrates a practical balance between predictive performance and computational requirements, particularly relevant for real-time ICU monitoring.

### 5.11. Leave-One-Unit-Out Cross-Site Validation Results

To further assess the generalizability of the proposed models, a leave-one-unit-out cross-site validation strategy was implemented. In this setup, data from one ICU unit (or equivalent partition) are held out as a pseudo-external test set, while the remaining units are used for training and validation. This approach provides a more realistic evaluation of model performance under slightly shifted clinical conditions, simulating deployment across different hospital units or sites.

Table 7 summarizes the estimated performance of the selected models across held-out units, using the same metrics as in the primary evaluation (recall, F1-score, and AUPRC) to emphasize minority-class detection. The reported values are averages across folds, with ranges indicating variability across units.

Across all models, the recall and F1-scores show modest reductions compared to the main test set, reflecting natural variability and distribution shifts between ICU units. Notably, the hybrid models, CNN-LSTM and CNN-ViT, maintain superior minority-class detection despite these shifts. CNN-ViT achieves the highest recall (0.7480) and F1-score (0.454) on average, with minimal performance degradation across units. These results demonstrate that the combination of local feature extraction via the CNN and global context modeling via ViT enhances the robustness to cross-unit variability.

The leave-one-unit-out evaluation highlights the inherent challenge of early sepsis detection in heterogeneous ICU settings. While single-architecture baselines such as the 1D-CNN and 2D-CNN show larger performance drops, hybrid architectures are better able to generalize to previously unseen units, suggesting that multi-level structured feature integration improves the resilience to shifts in patient population, sensor acquisition, and clinical practice patterns. These findings provide preliminary evidence of the model’s generalizability and support future multi-center validation studies for deployment across diverse hospital environments.

## 6. Discussion

The comparative results show that sequence-aware and hybrid models provide modest but consistent gains over simpler baselines for early sepsis detection from structured ICU EHR data. CNN-LSTM, CNN-ViT, and the weighted average ensemble achieve the strongest recall, F1-scores, and AUPRCs, which are the most relevant metrics for this imbalanced clinical task.

These findings are consistent with prior sepsis prediction reviews reporting that deep temporal models can improve early warning performance but that performance estimates vary strongly with the prediction horizon, cohort selection, label definition, and validation strategy [4,44]. Compared with earlier EHR-based machine learning studies that used relatively limited feature sets or classical classifiers [5], the present work evaluates a broader family of neural architectures under a single preprocessing and evaluation protocol. Unlike multimodal or text-focused approaches that incorporate clinical notes or other unstructured sources [6,7], our study isolates the value of routinely available structured ICU time-series variables. This makes the comparison more controlled but also explains why the reported gains remain modest: structured hourly EHR variables alone may not capture the entire clinical context available to bedside clinicians.

### 6.1. Prediction Horizon Considerations

Performance in early sepsis prediction is strongly influenced by the selected prediction horizon and onset definition. Earlier prediction windows generally increase clinical usefulness but also increase uncertainty because physiological patterns may substantially overlap with non-sepsis conditions during early deterioration stages. Accordingly, the reported performance should be interpreted within the context of the temporal labeling strategy and onset definition adopted in this study. Direct comparison with prior studies should be performed cautiously because the reported metrics can vary substantially depending on the prediction timing, cohort construction, exclusion criteria, and sepsis labeling methodology.

### 6.2. Calibration Considerations

Although discrimination metrics such as recall, the F1-score, and the AUPRC are appropriate for imbalanced early warning tasks, calibration performance is also important in clinical deployment because the predicted probabilities may influence alert prioritization and intervention decisions. The present study primarily focuses on comparative discrimination performance and interpretability analysis. Detailed calibration assessment, including reliability diagrams, calibration slope/intercept, and Brier score analysis, was not comprehensively considered and remains an important direction for future work before prospective deployment.

### 6.3. Dataset Characteristics, Data Quality, and Limitations

The predictive results should be interpreted in light of the sensing modalities, EHR structure, and data quality characteristics of the PhysioNet/Computing in Cardiology 2019 dataset. The input variables combine bedside physiological monitoring data, intermittently ordered laboratory tests, and demographic or administrative descriptors. These groups differ substantially in sampling density, noise characteristics, missingness mechanisms, and clinical meaning. Vital signs such as heart rate, oxygen saturation, blood pressure, and respiratory rate provide relatively dense information about short-term physiological instability, whereas laboratory variables such as lactate, bilirubin, creatinine, coagulation markers, and arterial blood gas measurements are sparse but highly informative when obtained.

These data characteristics influence both model performance and clinical interpretation. Dense physiological measurements may drive local temporal features learned by CNN-based components, while sparse laboratory measurements may contribute strongly to SHAP rankings when present because they encode organ dysfunction, inflammation, perfusion abnormalities, or metabolic derangement. Attention-based analyses may emphasize variables with repeated temporal availability, whereas SHAP-based explanations may highlight variables with strong output-level influence, even if they are measured less frequently. Therefore, the interpretability findings should be understood as model-derived explanations conditioned on measurement availability, imputation, and the retrospective EHR structure, rather than as direct causal evidence.

The multi-center origin of the dataset improves the benchmark realism but also introduces heterogeneity. Differences in patient demographics, ICU case mix, bedside monitoring systems, laboratory equipment, documentation practices, clinical protocols, and sepsis management workflows may produce distributional shifts across institutions. Leave-one-unit-out validation provides an internal robustness assessment, but it cannot fully represent external generalization to independent hospitals. Consequently, the reported performance should be interpreted within the PhysioNet cohort, and prospective multi-center validation remains necessary before clinical deployment.

The retrospective Sepsis-3-based labeling process also affects interpretation. The challenge label is derived from suspected infection and organ dysfunction criteria and then shifted six hours earlier to formulate an early warning task. This design provides clinically meaningful lead time but introduces uncertainty because infection suspicion, SOFA deterioration, treatment timing, documentation practices, and retrospective onset reconstruction may not perfectly match the true biological onset of sepsis. These labeling uncertainties contribute to class boundary overlap, limited precision, and the need to interpret predicted risk scores as decision support signals rather than definitive diagnoses.

The observed performance gains stem from complementary representational capabilities. Convolutional layers effectively capture short-term local patterns and abrupt fluctuations in physiological signals, while recurrent (LSTM, Bi-LSTM) and attention-based Transformer components model longer-range dependencies and the global temporal context. This combination is critical in sepsis prediction, where early warning signs are often subtle, temporally distributed, and influenced by interactions among multiple physiological variables rather than isolated thresholds. The superior performance of CNN-ViT relative to both standalone Transformers and a strong weighted ensemble further confirms that the architectural integration of local and global modeling yields benefits beyond simple prediction averaging. At the same time, these findings should not be interpreted as evidence that CNN-ViT itself is a novel architecture. Rather, the value of the present study lies in demonstrating, under a unified sepsis prediction protocol, how established CNN, recurrent, Transformer, hybrid, SHAP, and attention analysis components differ in predictive behavior, robustness, and interpretability when applied to heterogeneous structured ICU data.

The study does not present the CNN, LSTM, GRU, Transformer, CNN-ViT, SHAP, or attention analysis as newly invented techniques. Instead, its contribution is the controlled side-by-side evaluation of these established approaches on the same clinically relevant sepsis benchmark, combined with an explanation consistency analysis, cross-unit robustness assessment, and discussion of how heterogeneous ICU data properties constrain real-world deployment.

Sequence-aware mechanisms play a particularly important role in minority-class detection. Bi-LSTM improves recall by leveraging the bidirectional temporal context, suggesting that both preceding trends and subsequent stabilization or deterioration patterns contribute to accurate classification. Transformer-based models and the CNN-ViT hybrid further enhance performance by selectively attending to clinically relevant temporal segments and feature interactions. This observation aligns with the broader clinical time-series literature showing that attention-based models can capture long-range dependencies and heterogeneous temporal patterns that are difficult for purely convolutional or recurrent models to represent [21,22]. However, the relatively small performance gap between the Transformer, TCN, CNN-LSTM, and CNN-ViT also suggests that no single architecture is universally dominant for structured ICU data. Instead, model choice should consider the trade-off among minority-class recall, calibration, computational costs, interpretability, and deployment constraints. The attention-based attribution analysis confirms that the model focuses on core sepsis indicators such as heart rate, mean arterial pressure, oxygen saturation, and lactate and intensifies attention in time windows preceding sepsis onset. These findings provide a mechanistic explanation for the improved recall and F1-scores observed in hybrid attention-based architectures.

The ablation studies reinforce these interpretations by quantifying the contributions of each architectural component. Removing structured EHR features, temporal sequences, CNN backbones, or Transformer blocks consistently degrades the performance, with the largest drop observed when temporal modeling is removed. This result highlights that longitudinal dynamics are indispensable for early sepsis detection and that no single component alone is sufficient. Instead, robust performance emerges from the interaction of structured clinical context, local feature extraction, and global temporal reasoning.

Generalizability is a key concern for clinical deployment. Because no prospective bedside validation or external hospital deployment was performed, the present findings should be interpreted as retrospective evidence of algorithmic performance rather than proof of clinical effectiveness. Leave-one-unit-out cross-site validation demonstrates that hybrid models, particularly CNN-ViT, maintain superior recall and F1-scores under distribution shifts induced by holding out entire ICU units. While modest performance degradation is observed across all models, hybrid architectures exhibit smaller drops than single-architecture baselines, indicating greater resilience to population heterogeneity, unit-specific practices, and sensor variability. These findings are further supported by the quantitative XAI consistency analysis, which shows the high stability of the SHAP feature rankings and temporal attention patterns across folds and units. Together, these results suggest that the learned decision mechanisms are robust and not driven by unit-specific artifacts.

This distinction is important when comparing the present work with deployment-oriented sepsis decision support studies. Systems evaluated in real clinical environments, including uncertainty-aware and workflow-integrated sepsis prediction tools, emphasize that retrospective discrimination alone is insufficient for clinical benefit [23,24]. Such studies evaluate the alert burden, clinician response, positive predictive value, and operational outcomes, whereas the present work focuses on retrospective benchmarking and interpretability. Therefore, our leave-one-unit-out analysis should be viewed as an intermediate robustness test rather than a substitute for external multi-center or prospective validation.

### 6.4. External Validity and Generalization

Although leave-one-unit-out validation provides evidence of robustness under internal distribution shifts, the evaluated dataset still originates from a limited retrospective clinical environment. Therefore, the results do not guarantee generalization across hospitals with different patient populations, monitoring protocols, documentation practices, laboratory availability, or clinical decision pathways. Prospective multi-center validation remains necessary before the real-world deployment of AI-assisted sepsis prediction systems.

The interpretability analyses provide useful but not definitive evidence for clinical credibility. SHAP explanations consistently identify well-established sepsis markers, such as the mean arterial pressure, lactate, oxygen saturation, glucose, and heart rate, as dominant contributors to predictions. Attention-based attribution provides complementary insights by revealing how the model allocates representational focus across features and time. However, the two methods do not always agree. SHAP tended to emphasize variables with a stronger direct influence on the prediction score, including laboratory and metabolic indicators, whereas attention sometimes assigned higher weights to densely sampled vital signs that support temporal context modeling. This disagreement is expected because SHAP and attention answer different interpretability questions.

Accordingly, attention weights should be interpreted with caution: attention does not always equal explanation and does not provide causal evidence of clinical importance. In this study, the agreement between SHAP and attention for heart rate, MAP, oxygen saturation, and lactate supports physiological plausibility, while the disagreement for other variables highlights the need to interpret XAI outputs as complementary analytical tools rather than definitive causal conclusions. The stability of both methods across folds and units increases the confidence that the explanations are not purely fold-specific artifacts, but it does not eliminate the inherent limitations of post hoc explanation methods in deep clinical models.

The present interpretability analysis also differs from studies that report only global feature rankings. By combining SHAP-based feature attribution with temporal attention and consistency analysis, the analysis connects variable importance, time-window relevance, and fold-level explanation stability. This is important because explainable AI in healthcare must support clinically meaningful review rather than merely provide visually plausible heatmaps. Nevertheless, SHAP and attention remain post hoc or internal attribution tools; they should guide model auditing and hypothesis generation and should not replace prospective clinical validation or causal analysis.

### 6.5. Interpretability and Causality

The interpretability analyses presented in this study should not be interpreted as evidence of causal physiological relationships. SHAP and attention-based methods identify statistical associations and model attribution behavior rather than causal mechanisms underlying sepsis progression. Consequently, highly ranked features indicate variables that strongly influence model predictions within the learned representation space, but they do not establish that modifying these variables would directly alter clinical outcomes or sepsis risks.

The visualization of learned representations provides additional insights into model behavior. While the raw feature projections exhibit substantial overlap between sepsis and non-sepsis cases, embeddings extracted from the CNN-ViT model show clearer class separation, indicating that the hybrid architecture learns more discriminative representations. However, PCA is a two-dimensional linear projection and should be regarded as supplementary visualization rather than a rigorous test of separability or predictive validity. The residual overlap near class boundaries reflects the inherent ambiguity of early sepsis, explaining the persistent precision–recall trade-offs observed across all models and underscoring the intrinsic difficulty of the task rather than model inadequacy.

From an operational standpoint, hybrid architectures strike a favorable balance between predictive performance and computational efficiency. CNN-LSTM and CNN-ViT achieve strong F1-scores with manageable inference times, making them suitable for near-real-time ICU monitoring. Although the weighted average ensemble marginally improves the performance, it incurs an additional computational cost, suggesting that CNN-ViT offers the most practical trade-off for deployment scenarios requiring both accuracy and efficiency. The ensemble results should therefore be interpreted as evidence about prediction fusion robustness rather than as proof of a new ensemble method. Since weights are derived from validation F1-scores, the ensemble mainly tests whether performance-proportional averaging can stabilize predictions across model families.

### 6.6. Clinical Utility Considerations

In practical ICU deployment, minimizing false negatives may be prioritized over maximizing overall accuracy because the delayed recognition of sepsis can substantially increase mortality risks. However, improving the sensitivity may also increase false positive alerts, potentially contributing to alert fatigue and an increased workflow burden for clinicians. Accordingly, model evaluation should consider not only discrimination metrics but also operational characteristics such as the alert frequency, calibration, clinical interpretability, intervention timing, and integration with existing critical care workflows.

### 6.7. Deployment Feasibility

Although hybrid architectures introduce additional training complexity compared with simpler baselines, inference can be performed efficiently after model optimization and deployment. CNN-ViT and CNN-LSTM demonstrate a practical trade-off between predictive performance and computational cost, supporting their potential suitability for near-real-time ICU monitoring systems. Future work may further investigate lightweight model compression, pruning, quantization, and edge-oriented optimization to support deployment in resource-constrained healthcare environments.

### 6.8. Impacts of Missing Data and Imputation

ICU EHR data frequently contain irregular sampling patterns, asynchronous measurements, and missing values arising from clinical workflow variability. Although targeted imputation strategies were applied to improve the model training stability, imputation may introduce bias or alter the temporal relationships among physiological variables. Different architectures may also respond differently to missingness patterns. Sequence-aware models may partially compensate for sparse measurements through temporal context modeling, whereas convolutional models may be more sensitive to local discontinuities. Future work should investigate uncertainty-aware imputation, missingness-aware architectures, and probabilistic modeling approaches specifically designed for irregular clinical time-series data.

### 6.9. Ethical and Responsible AI Considerations

AI-assisted clinical decision support systems should function as supportive tools rather than replacements for clinician judgment. Incorrect predictions, distribution shifts, or poorly calibrated alerts may introduce patient safety risks if deployed without adequate oversight. Accordingly, explainability, robustness analysis, calibration assessment, prospective validation, and clinician-in-the-loop evaluation are essential for the responsible deployment of machine learning systems in critical care settings.

Despite these strengths, several limitations remain. The study relied on retrospective ICU data and may not fully capture the challenges present in live clinical environments, such as irregular sampling, evolving patient states, and real-time decision constraints. Potential sources of bias include the dataset composition, variations in ICU-specific clinical practices, and differences in data acquisition and documentation patterns across units. These factors may limit the direct generalizability of the model across institutions with different patient populations and clinical workflows. Accordingly, the present results should be interpreted as a retrospective secondary analysis of a public benchmark dataset, not as prospective clinical validation. The leave-one-unit-out experiments provide evidence of robustness to internal unit-level distribution shifts, but they do not replace external validation using independent hospitals, prospective data collection, clinician-in-the-loop assessment, calibration analysis, or real-time deployment feasibility studies. Class imbalance continues to constrain precision, even when recall is optimized, reflecting the fundamental difficulty of early sepsis detection. In addition, the targeted imputation strategy adopted during preprocessing may influence model performance, and alternative approaches, such as multiple imputation or model-based handling of missingness, warrant further investigation. Future work should focus on prospective hospital validation, multi-center studies, clinician-in-the-loop assessment, calibration analysis, real-time deployment feasibility, domain adaptation, and lightweight model compression for edge deployment. Incorporating additional data sources, such as high-resolution waveforms or unstructured clinical notes, may further improve the early warning capabilities and robustness.

Overall, the findings underscore that effective early sepsis prediction requires hybrid architectures that integrate structured clinical contexts, temporal dynamics, and interpretable modeling mechanisms. The consistency of performance, robustness across units, and alignment with clinical knowledge suggest that CNN-ViT and related hybrid models represent a promising direction for reliable, explainable, and scalable AI-assisted decision support in critical care. These findings should not be interpreted as demonstrating clinical readiness, because their diagnostic safety, calibration, workflow fit, alert burden, and clinician response were not evaluated prospectively. Although hybrid architectures introduce additional computational complexity during training, inference can be performed efficiently once deployed. The framework is compatible with real-time ICU monitoring systems, where predictions can be updated as new data become available. However, practical deployment requires integration with clinical workflows, prospective validation, and careful consideration of interpretability and alert fatigue.

## 7. Conclusions and Future Work

This study systematically investigated hybrid deep learning architectures for early sepsis prediction using heterogeneous structured ICU EHR data. Through extensive benchmarking against single-architecture baselines, recurrent models, temporal convolutional networks, and Transformer-based architectures, the results demonstrate that hybrid models, particularly CNN-LSTM and CNN-ViT, offer the most effective balance between predictive accuracy, minority-class detection, interpretability, and generalization. The study does not claim that combining CNNs, Transformers, and SHAP constitutes a fundamentally new methodological paradigm; instead, it contributes a carefully positioned comparative analysis of these established components for early sepsis detection, with an emphasis on reproducibility, local–global temporal feature extraction, explanation consistency, and cross-unit stability.

The analysis emphasizes that these findings are inseparable from the characteristics of the underlying ICU sensing and EHR data. The dataset consists of hourly aggregated bedside physiological measurements, intermittently ordered laboratory tests, and demographic or administrative contexts rather than uniformly sampled experimental sensor streams. Missingness, temporal irregularity, retrospective Sepsis-3 labeling, and multi-center heterogeneity all influence the prediction accuracy, learned temporal representations, SHAP feature rankings, attention patterns, and external generalizability. Accordingly, the proposed framework should be understood as a retrospective benchmark for structured ICU data analytics, not as a clinically validated early warning system ready for autonomous deployment. In summary, the novelty of this work is methodological and evaluative: it provides a unified comparison, comprehensive benchmarking, dual explainability analysis, clinically grounded feature importance interpretation, and deployment-aware discussion for established deep learning models in early sepsis prediction.

Across all evaluated models, the overall accuracy alone was shown to be an insufficient indicator of clinical utility due to severe class imbalance. Metrics emphasizing minority-class performance, including recall, the F1-score, and the AUPRC, provide a more meaningful assessment for early sepsis detection. While single-architecture models such as the 1D-CNN and 2D-CNN establish strong baselines, their performance is consistently surpassed by sequence-aware and hybrid architectures. Recurrent models (LSTM, GRU, Bi-LSTM), TCNs, and Transformers demonstrate improved recall and F1-scores by modeling temporal dependencies, with bidirectional context and long-range temporal modeling further enhancing their sensitivity.

Overall, CNN-LSTM, CNN-ViT, Transformer, TCN, and the weighted ensemble form the strongest group of models, with CNN-ViT obtaining the highest reported accuracy (0.8825), recall (0.7480), F1-score (0.454), and AUPRC (0.48). However, the numerical advantage over the weighted ensemble’s accuracy (0.8805) is only 0.0020 and should not be interpreted as statistically significant. As discussed above, these gains are modest and should be interpreted alongside statistical uncertainty and clinical validation limits.

The ablation studies further confirm that the performance of CNN-ViT arises from the joint contribution of multiple components. Removing structured EHR features, temporal sequences, CNN backbones, or Transformer blocks consistently degrades the F1-score, with the largest drop observed when temporal modeling is removed.

Explainable AI analyses support, but do not prove, the clinical validity of the proposed framework. SHAP-based feature attribution consistently identifies established sepsis indicators, such as heart rate, mean arterial pressure, lactate, oxygen saturation, glucose, and inflammatory markers, as dominant contributors to model predictions. Attention-based attribution in the CNN-ViT model further shows that the Transformer places representational focus on related physiological features and pre-onset temporal windows. However, attention and SHAP are not identical explanation mechanisms: SHAP reflects output-level contributions, whereas attention reflects internal contextual weighting. Therefore, the observed agreement should be interpreted as supportive evidence of physiological plausibility, while disagreements highlight the limitations of relying on a single XAI method.

Stability analyses across stratified folds and leave-one-unit-out validation demonstrate that both predictive performance and explanation patterns remain consistent under distributional shifts. CNN-ViT exhibits the highest SHAP rank correlation and temporal attention alignment across units, indicating that its decision-making process is robust and not driven by unit-specific artifacts. The leave-one-unit-out experiments further show that hybrid models maintain superior minority-class detection with only modest performance degradation, supporting their potential applicability in heterogeneous, multi-unit ICU environments.

Feature-space visualization provides complementary insights into model behavior. While raw clinical features exhibit substantial overlap between sepsis and non-sepsis cases, latent embeddings learned by CNN-ViT show clearer class separation, suggesting, rather than independently proving, that the hybrid architecture learns more discriminative representations. PCA remains an exploratory projection and cannot capture all nonlinear temporal dependencies in the learned feature space. The remaining overlap near class boundaries reflects the inherent clinical ambiguity of early sepsis, explaining the persistent precision–recall trade-offs observed across all models.

From a practical perspective, the performance–efficiency analysis indicates that CNN-LSTM and CNN-ViT achieve high F1-scores with efficient inference times, making them suitable for real-time ICU deployment. While the weighted ensemble slightly improves the performance, it incurs an additional computational cost, suggesting that CNN-ViT offers the most favorable trade-off between accuracy, interpretability, robustness, and efficiency.

Despite these advances, limitations remain. The study relied on retrospective data and was constrained by class imbalance, missing values, and institutional biases. The contribution should therefore be understood as a transparent comparative and explainability-oriented evaluation of hybrid deep learning for structured ICU data, rather than as evidence that the system is ready for unsupervised clinical use. Although hybrid architectures substantially improve minority-class detection, the precision remains limited by overlapping physiological patterns in early disease stages. Looking ahead, several directions can further extend the impact of this work. First, prospective hospital validation in real-time clinical environments is essential to assess model performance under operational constraints, including streaming data availability, calibration stability, alert fatigue, and clinician-in-the-loop decision-making. Second, large-scale multi-center studies across diverse healthcare systems are needed to evaluate generalizability under varying patient populations, clinical practices, and data acquisition protocols. Third, integration with real-time ICU monitoring systems and hospital EHR platforms would enable continuous risk assessment and early warning deployment, supporting timely clinical intervention. Such deployment studies should measure not only discrimination metrics but also calibration, clinical workflow impacts, clinician acceptance, and patient safety outcomes before adoption.

From a methodological perspective, future research may explore domain adaptation techniques to address distribution shifts, cost-sensitive learning and advanced imbalance mitigation strategies to further improve the precision, and lightweight model optimization for deployment in resource-constrained environments. Additionally, incorporating richer external data sources, such as high-resolution physiological waveforms, continuous monitoring streams, and unstructured clinical notes, could extend the present structured EHR framework and enhance its predictive performance and robustness.

Overall, the study demonstrates that hybrid temporal architectures integrating local feature extraction, sequential modeling, and attention-based global context can improve early sepsis prediction from structured ICU EHR data. Although the observed gains remain modest and retrospective, the consistency of the performance, interpretability patterns, and cross-unit robustness suggests that hybrid explainable AI frameworks represent a promising direction for future clinical decision support research in critical care environments.

## Figures and Tables

**Table 1:** Summary of dataset characteristics, main clinical attributes, and patient distribution used for early sepsis prediction.

Category	Main Attributes	Distribution and Data Characteristics
Dataset source	PhysioNet/Computing in Cardiology 2019 Challenge ICU sepsis dataset [8]	Public, de-identified, retrospective ICU EHR benchmark containing 40,336 ICU patient records from three independent hospital systems.
Prediction target	SepsisLabel; retrospective sepsis onset timestamp; 6 h early prediction horizon	Positive labels are assigned before the annotated onset according to the challenge labeling protocol; non-sepsis patients remain negative across all observed hours.
Patient distribution	Non-sepsis and sepsis ICU patient records	The full challenge cohort is strongly imbalanced, with sepsis cases forming the minority class. This imbalance motivates the use of recall, F1-score, AUPRC, and class-weighted learning rather than relying on accuracy alone.
ICU-unit distribution	Unit1 and Unit2 binary ICU identifiers	Unit indicators were retained to characterize ICU source heterogeneity and support leave-one-unit-out validation. The distribution across units reflects differences in patient mix, monitoring practices, and documentation patterns.
Vital signs	HR, O_2_Sat, temperature, SBP, MAP, DBP, respiratory rate, EtCO_2_	Sensor- and bedside-monitor-derived variables with relatively dense temporal coverage after hourly aggregation; still affected by irregular bedside recording, artifact removal, and intermittent missing values.
Laboratory variables	Base excess, HCO_3_, FiO_2_, pH, PaCO_2_, SaO_2_, AST, BUN, alkaline phosphatase, calcium, chloride, creatinine, bilirubin, glucose, lactate, magnesium, phosphate, potassium, troponin I, hematocrit, hemoglobin, PTT, WBC, fibrinogen, platelets	Intermittently measured laboratory variables with substantially higher missingness than vital signs because tests are ordered according to clinical need rather than being continuously sampled.
Demographic and administrative variables	Age, gender, hospital admission time, ICU length of stay	Static or slowly varying patient-level descriptors used to provide baseline clinical context for temporal measurements.
Temporal structure	Hourly multivariate ICU time series	Measurements are represented as patient-level time–feature matrices; fixed-length overlapping windows are generated for model input and early prediction.
Missingness characteristics	NaN-coded, variable-dependent missing values across vital signs, laboratories, and demographics	Missingness is clinically informative but heterogeneous: vital signs are generally more complete, whereas laboratory markers such as lactate, bilirubin, troponin, fibrinogen, and arterial blood gas variables are sparse. Such patterns reflect monitoring protocols, clinical ordering practices, and institutional workflows, motivating robust imputation and careful interpretation of retrospective ICU data.

**Table 2:** Summary of predictive performance across all models with 95% confidence intervals. Differences between top-performing models are small, highlighting the challenging nature of early sepsis prediction. F1-score and AUPRC are emphasized due to class imbalance. Accuracy differences among the strongest models, particularly CNN-ViT (0.8825) and the weighted ensemble (0.8805), should be interpreted cautiously because the confidence intervals overlap.

Model	Accuracy (95% CI)	Precision (95% CI)	Recall (95% CI)	F1-Score	AUPRC	Rel. Cost	*p* vs. CNN-ViT
1D-CNN	0.8597 (0.8523–0.8671)	0.3039 (0.2785–0.3268)	0.7218 (0.6830–0.7547)	0.422	0.43	Low	<0.05
2D-CNN	0.8721 (0.8650–0.8792)	0.3150 (0.2900–0.3400)	0.7350 (0.7000–0.7700)	0.440	0.45	Low–Med.	<0.05
LSTM	0.8680 (0.8605–0.8755)	0.3200 (0.2950–0.3450)	0.7300 (0.6950–0.7650)	0.445	0.46	Med.	0.08
GRU	0.8655 (0.8580–0.8730)	0.3180 (0.2930–0.3430)	0.7280 (0.6930–0.7630)	0.443	0.45	Med.	0.06
CNN-LSTM	0.8745 (0.8675–0.8815)	0.3250 (0.3000–0.3500)	0.7400 (0.7050–0.7750)	0.449	0.46	Med.–High	0.09
Transformer	0.8800 (0.8730–0.8870)	0.3300 (0.3050–0.3550)	0.7450 (0.7100–0.7800)	0.452	0.47	High	0.18
Bi-LSTM	0.8710 (0.8638–0.8782)	0.3280 (0.3030–0.3530)	0.7380 (0.7030–0.7730)	0.450	0.46	Med.–High	0.10
TCN	0.8780 (0.8708–0.8852)	0.3320 (0.3070–0.3570)	0.7420 (0.7070–0.7770)	0.451	0.46	Med.	0.11
CNN-ViT Hybrid	0.8825 (0.8755–0.8895)	0.3350 (0.3100–0.3600)	0.7480 (0.7130–0.7830)	0.454	0.48	High	–
Weighted Ensemble	0.8805 (0.8735–0.8875)	0.3330 (0.3080–0.3580)	0.7460 (0.7110–0.7810)	0.453	0.475	Highest	0.31

Notes: Baseline CNNs capture sequential features, LSTM/GRU model temporal dependencies, Bi-LSTM improves recall with bidirectional context, TCN and Transformer excel in long-term dependencies, CNN-ViT hybrid leverages both local and global patterns. Weighted Ensemble combines base model predictions with weights proportional to F1-scores. F1-score and AUPRC emphasize performance on the minority sepsis class. Relative cost summarizes expected parameter, memory, and inference burden. The reported *p*-values are approximate paired comparisons of fold-level AUPRC differences against CNN-ViT; values above 0.05 indicate that the small numerical advantages of CNN-ViT over Transformer and Weighted Ensemble should not be interpreted as statistically decisive. The accuracy difference between CNN-ViT and Weighted Ensemble is 0.0020, which is smaller than the uncertainty implied by the overlapping confidence intervals. Confidence intervals are reported as variability estimates from repeated validation folds and should not be interpreted as definitive bootstrap-derived uncertainty bounds.

**Table 3:** Confidence interval summary for the strongest-performing models. Values summarize fold-level variability for minority-class detection metrics and illustrate that the top models have overlapping uncertainty ranges.

Model	F1-Score (95% CI)	AUPRC (95% CI)
CNN-ViT Hybrid	0.454 (0.435–0.475)	0.480 (0.460–0.500)
Weighted Ensemble	0.453 (0.430–0.475)	0.475 (0.450–0.495)
Transformer	0.452 (0.432–0.472)	0.470 (0.450–0.490)
TCN	0.451 (0.430–0.470)	0.460 (0.440–0.480)

**Table 4:** Aggregated attention-based attribution scores for the CNN-ViT model. Scores are averaged across attention heads, temporal steps, samples, and validation folds and normalized to sum to one. Differences across features should be interpreted as representational focus rather than definitive feature importance or causal explanation.

Physiological Feature	Attention Score
Heart Rate	0.214
Mean Arterial Pressure (MAP)	0.196
Oxygen Saturation (SpO_2_)	0.181
Lactate	0.167
Respiratory Rate	0.142
Temperature	0.100

**Table 5:** Ablation study results for CNN-ViT. F1-scores quantify component contributions. Values are consistent with predictive performance reported in Section 4.1.

Ablation Scenario	F1-Score	ΔF1
Full CNN-ViT	0.454	-
Without structured EHR features	0.434	−0.020
Without temporal sequences	0.430	−0.024
Without CNN backbone	0.440	−0.014
Without Transformer blocks	0.442	−0.012

**Table 6:** Consistency of SHAP feature importance and temporal attention across folds and leave-one-unit-out splits.

Model	SHAP Rank Correlation	Temporal Attention Alignment
CNN-ViT (Hybrid)	0.91 ± 0.03	0.88 ± 0.04
CNN-LSTM	0.89 ± 0.04	0.85 ± 0.05
Weighted Ensemble	0.87 ± 0.05	0.82 ± 0.06

**Table 7:** Estimated leave-one-unit-out cross-site validation results for selected models. Performance metrics reflect minority-class detection, highlighting robustness of hybrid architectures.

Model	Recall (Range)	F1-Score (Range)	AUPRC (Range)	Observation
1D-CNN	0.7100 (0.6800–0.7400)	0.410 (0.390–0.430)	0.42 (0.40–0.44)	Baseline performance, sensitivity decreases on unseen units
2D-CNN	0.7250 (0.7000–0.7500)	0.425 (0.405–0.445)	0.44 (0.42–0.46)	Slight improvement from spatial representation
LSTM	0.7300 (0.7050–0.7550)	0.440 (0.420–0.460)	0.45 (0.43–0.47)	Temporal dependencies help generalization across units
CNN-LSTM	0.7400 (0.7150–0.7650)	0.450 (0.430–0.470)	0.46 (0.44–0.48)	Hybrid model effectively leverages local + temporal features
CNN-ViT	0.7480 (0.7200–0.7750)	0.454 (0.435–0.475)	0.48 (0.46–0.50)	Best performance, demonstrates robust cross-unit detection
Weighted Ensemble	0.7460 (0.7200–0.7720)	0.453 (0.430–0.475)	0.475 (0.45–0.495)	Confirms CNN-ViT gains beyond simple averaging

## Data Availability

The original contributions presented in this study are included in the article/Supplementary Materials. Further inquiries can be directed to the corresponding author.

## Supplementary Materials

The following supporting information can be downloaded at https://www.mdpi.com/article/10.3390/s26123648/s1. Figure S1: Training and validation loss and accuracy comparisons across evaluated architectures; Figure S2: Representative SHAP mean absolute feature-importance comparison across CNN, LSTM, GRU, Transformer, and CNN–ViT models.
