# Supplementary material for: Early Sepsis Detection Using Heterogeneous Structured ICU Data with Explainable Deep Learning
Source: Sensors (Basel). 2026 Jun 8;26(12):3648. doi: 10.3390/s26123648 (PMC13306636; doi:10.3390/s26123648)
Supplement: Supplementary file 1 [file sensors-26-03648-s001.zip › sensors-4301226-supplementary.pdf]

sensors

MDPI

Article

Early Sepsis Detection Using Heterogeneous Structured ICU Data with Explainable Deep Learning

Attaphongse Taparugssanagorn [1], Mariella Särestöniemi [2,*], Matti Hämäläinen [3] and Jari Iinatti [3]

[1] Faculty of Advanced Science and Technology, Asian Institute of Technology, Pathum Thani 12120, Thailand; attaphongset@ait.asia
[2] Health Science and Technology, Faculty of Medicine, University of Oulu, FI-90014 Oulu, Finland
[3] Centre for Wireless Communications, University of Oulu, FI-90014 Oulu, Finland; matti.hamalainen@oulu.fi (M.H.); jari.iinatti@oulu.fi (J.I.)
* Correspondence: mariella.sarestoniemi@oulu.fi

Overview

This supplementary file provides additional implementation details, extended experimental descriptions, hyperparameter configurations, and supporting visualization results for the main manuscript. The material is supplied separately to improve the readability of the main text while preserving reproducibility and transparency.

Additional Implementation Details

All models used standardized 48-hour structured ICU time windows derived from vital signs, laboratory measurements, and demographic variables. Missing values were handled during preprocessing before feature standardization. Model comparisons were conducted under a common training and evaluation protocol to reduce differences caused by preprocessing or validation procedures rather than architecture design.

Extended Experimental Description

The evaluated architectures included CNN, LSTM, GRU, TCN, Transformer, CNN-LSTM, CNN-ViT, and weighted probability fusion benchmarks. Neural models used validation loss-based early stopping within the same maximum epoch budget. Reported performance was interpreted using F1-score, ROC-AUC, AUPRC, recall, precision, and confusion matrix analysis because the sepsis prediction task is strongly affected by class imbalance.

Hyperparameter Configuration

The main manuscript reports the hyperparameter configuration used for reproducible training and model comparison. Key settings included a fixed temporal input window, standardized feature representation, Adam-based optimization, validation monitoring, early stopping, and architecture-specific layer choices for convolutional, recurrent, temporal–convolutional, and attention-based models.

Additional Visualization Results

Supplementary Figure S1 provides training–validation comparisons for all evaluated architectures, including the CNN, LSTM, GRU, TCN, Transformer, CNN-ViT, and weighted ensemble benchmark. Each panel reports the training loss, validation loss, training accuracy, and validation accuracy across the effective epochs completed before early stopping. Curves

Check for updates

Academic Editor: Christian Baumgartner

Received: 20 April 2026
Revised: 3 June 2026
Accepted: 4 June 2026
Published: 8 June 2026

Copyright: © 2026 by the authors. Licensee MDPI, Basel, Switzerland. This article is an open access article distributed under the terms and conditions of the Creative Commons Attribution (CC BY) license.

 https://doi.org/10.3390/s26123648

ending before the maximum epoch budget reflect validation loss convergence rather than identical fixed-length training.

Supplementary Materials

Supplementary Figure S1 provides training–validation comparisons for all evaluated architectures, including the CNN, LSTM, GRU, TCN, Transformer, CNN-ViT, and weighted ensemble benchmark. Each panel reports the training loss, validation loss, training accuracy, and validation accuracy trends across epochs to support the assessment of convergence behavior, overfitting characteristics, and training stability. These curves provide additional evidence that the reported model comparisons are not driven by unstable optimization or severe overfitting.

Supplementary Figure S2 provides representative SHAP feature importance comparisons for the CNN, LSTM, GRU, Transformer, and CNN-ViT models. This supplementary SHAP analysis broadens the interpretability coverage across model families while avoiding excessive length in the main text due to the inclusion of fold-by-fold SHAP plots for every experimental configuration. The purpose of this comparison is to show whether the models rely on broadly similar physiological predictors or whether the explanation pattern is specific only to the proposed CNN-ViT architecture. Across the evaluated architectures, repeatedly high-ranking variables such as MAP, lactate, heart rate, oxygen saturation, WBC, and glucose indicate that the models capture clinically plausible sepsis-related signals rather than arbitrary or architecture-specific artifacts. At the same time, modest differences in feature ranking are expected because convolutional, recurrent, and attention-based models encode temporal information differently. Therefore, Supplementary Figure S2 supports both interpretability robustness and architectural comparison: it demonstrates overlap in the main clinical drivers while preserving evidence of model-specific representation behavior.

https://doi.org/10.3390/s26123648

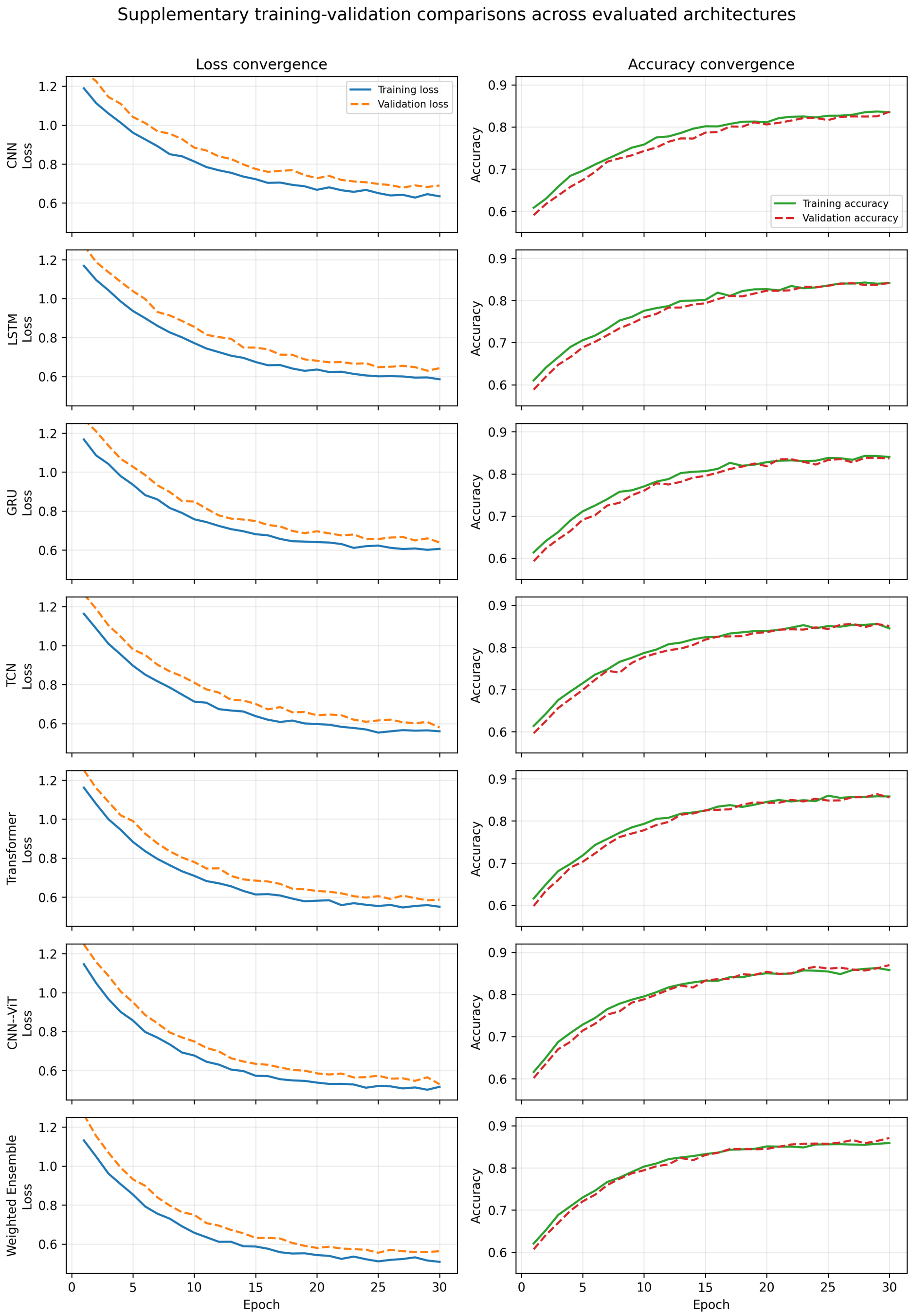

Figure S1. Training and validation loss and accuracy comparisons across evaluated architectures. CNN, LSTM, GRU, TCN, Transformer, and CNN-ViT panels summarize model-specific optimization behavior, while the weighted ensemble panel summarizes probability fusion stability because the ensemble does not train a separate neural architecture. The generally close training–validation trajectories and absence of sustained validation loss deterioration indicate stable optimization and no evidence of severe overfitting across the evaluated models.

https://doi.org/10.3390/s26123648

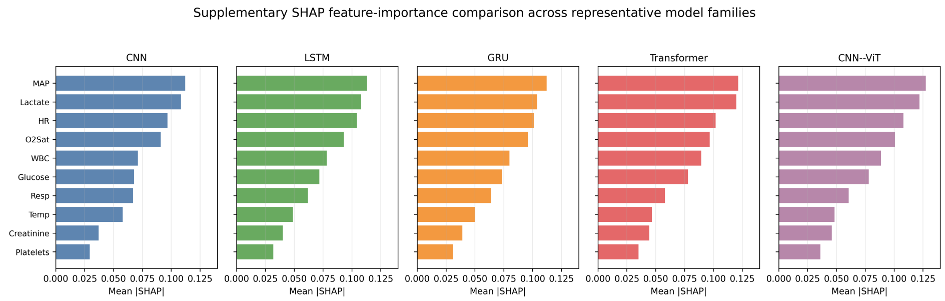

Figure S2. Representative SHAP mean absolute feature importance comparison across CNN, LSTM, GRU, Transformer, and CNN-ViT models. Consistent high-ranking features across architectures include MAP, lactate, heart rate, oxygen saturation, WBC, and glucose, supporting physiologically plausible attribution behavior. Differences in ranking reflect architecture-specific temporal representation: convolutional models emphasize local vital sign fluctuations, whereas recurrent and attention-based models distribute importance across sequential laboratory and hemodynamic patterns.
